# Neuronal and oligodendroglial, but not astroglial, tau translates to in vivo tau PET signals in individuals with primary tauopathies

**DOI:** 10.1007/s00401-024-02834-7

**Published:** 2024-11-24

**Authors:** Luna Slemann, Johannes Gnörich, Selina Hummel, Laura M. Bartos, Carolin Klaus, Agnes Kling, Julia Kusche-Palenga, Sebastian T. Kunte, Lea H. Kunze, Amelie L. Englert, Yunlei Li, Letizia Vogler, Sabrina Katzdobler, Carla Palleis, Alexander Bernhardt, Alexander Jäck, Andreas Zwergal, Franziska Hopfner, Sebastian N. Roemer-Cassiano, Gloria Biechele, Sophia Stöcklein, Gerard Bischof, Thilo van Eimeren, Alexander Drzezga, Osama Sabri, Henryk Barthel, Gesine Respondek, Timo Grimmer, Johannes Levin, Jochen Herms, Lars Paeger, Marie Willroider, Leonie Beyer, Günter U. Höglinger, Sigrun Roeber, Nicolai Franzmeier, Matthias Brendel

**Affiliations:** 1 Department of Nuclear Medicine, LMU Hospital, Ludwig Maximilian University of Munich, Marchioninstraße 15, 81377 Munich, Germany; 2 German Center for Neurodegenerative Diseases (DZNE) Munich, Munich, Germany; 3 Department of Neurology, LMU Hospital, Ludwig Maximilian University of Munich, Munich, Germany; 4 Munich Cluster for Systems Neurology (SyNergy), Munich, Germany; 5 German Center for Vertigo and Balance Disorders, DSGZ, LMU Hospital, Ludwig Maximilian University of Munich, Munich, Germany; 6 Department of Radiology, LMU Hospital, Ludwig Maximilian University of Munich, Munich, Germany; 7 Cognitive Neuroscience, Institute for Neuroscience and Medicine (INM-3), Research Centre Juelich, Juelich, Germany; 8 Department of Nuclear Medicine, University Hospital Cologne, Cologne, Germany; 9 Department of Neurology, University Hospital Cologne, Cologne, Germany; 10 German Center for Neurodegenerative Diseases (DZNE), Bonn, Germany; 11 Department of Nuclear Medicine, University Hospital Leipzig, Leipzig, Germany; 12 Department of Neurology, Medizinische Hochschule Hannover, Hannover, Germany; 13 Center for Cognitive Disorders, Department of Psychiatry and Psychotherapy, School of Medicine and Health, Technical University of Munich, Klinikum rechts der Isar, Munich, Germany; 14 Center of Neuropathology and Prion Research, Faculty of Medicine, LMU Munich, Munich, Germany; 15 Institute for Stroke and Dementia Research, LMU Hospital, LMU Munich, Munich, Germany; 16 Institute of Neuroscience and Physiology, Department of Psychiatry and Neurochemistry, , University of Gothenburg, The Sahlgrenska Academy, Mölndal, Gothenburg, Sweden

**Keywords:** Tau, PET, PI-2620, Autopsy, Autoradiography, Neuron

## Abstract

Tau PET has attracted increasing interest as an imaging biomarker for 4-repeat (4R)-tauopathy progressive supranuclear palsy (PSP). However, the translation of in vitro 4R-tau binding to in vivo tau PET signals is still unclear. Therefore, we performed a translational study using a broad spectrum of advanced methodologies to investigate the sources of [^18^F]PI-2620 tau PET signals in individuals with 4R-tauopathies, including a pilot PET autopsy study in patients. First, we conducted a longitudinal [^18^F]PI-2620 PET/MRI study in a 4-repeat-tau mouse model (PS19) and detected elevated [^18^F]PI-2620 PET signals in the presence of high levels of neuronal tau. An innovative approach involving cell sorting after radiotracer injection in vivo revealed higher tracer uptake in single neurons than in the astrocytes of PS19 mice. Regional [^18^F]PI-2620 tau PET signals during the lifetime correlated with the abundance of fibrillary tau and with autoradiography signal intensity in PSP patients and disease controls who underwent autopsy 2–63 months after tau PET. In autoradiography, tau-positive neurons and oligodendrocytes with a high AT8 density, but not tau-positive astrocytes, were the drivers of [^18^F]PI-2620 autoradiography signals in individuals with PSP. The high tau abundance in oligodendrocytes at the boundary of gray and white matter facilitated the identification of an optimized frontal lobe target region to detect the tau burden in patients with PSP. In summary, neuronal and oligodendroglial tau constitutes the dominant source of tau PET radiotracer binding in 4-repeat-tauopathies, translating to an in vivo signal.

**Supplementary Information:**

The online version contains supplementary material available at 10.1007/s00401-024-02834-7.

## Introduction

Progressive supranuclear palsy (PSP) is a rare neurodegenerative disease caused by the aggregation of tau protein with 4 microtubule-binding repeats [48], leading to death within 7–8 years after onset, on average [44]. In the past, the diagnosis was usually made 3–4 years after the first clinical symptoms, when patients already had severe functional disabilities. Controlled autopsy data revealed that the clinical diagnosis of PSP by the most recent MDS-PSP criteria has only limited sensitivity in the early stages with moderate specificity [42]. Currently, no approved causal therapy exists, and preventive and symptomatic treatment options are very limited. Since tau-targeting therapies are entering clinical development, diagnosing patients with PSP at an early stage is important to attenuate tau accumulation and therefore symptom progression. Furthermore, ensuring a high level of specificity for 4-repeat tau for inclusion in tau-targeting treatment trials is important to ensure adequate statistical power and to avoid unnecessary side effects on patients with clinically overlapping syndromes without 4-repeat tau aggregation. In this context, cohort studies in which the second-generation tau PET radiotracers [^18^F]PI-2620 and [^18^F]PM-PBB3 were used differentiated patients with PSP and CBS from healthy controls and disease controls [12, 39, 49]. However, head-to-head autoradiographic studies with different radiotracers by independent groups have produced inconsistent results, indicating the presence [32, 52] or absence of [1] binding of radiolabeled PI-2620 to 4R-tauopathy tissue sections. Furthermore, potential off-target sources still need to be considered for second-generation radiotracers, including neuromelanin and hemorrhagic lesions for [^18^F]PI-2620 [1] and β-amyloid for [^18^F]PM-PBB3 [26]. Nevertheless, competitive assays [52] and molecular docking studies [28] confirmed the initially reported affinity of PI-2620 for 4R-tau [27].

Hence, we aimed to investigate the translation of in vitro [^18^F]PI-2620 4R-tau binding to in vivo PET signals. We performed longitudinal 4R-tau monitoring in tau transgenic mice primarily expressing neuronal tau and pinpointed the cellular source of tracer signals by cell sorting after radiotracer injection in living organisms. In vivo PET signals in patients with definite PSP and disease controls were correlated with the tau abundance and autoradiography signals in autopsy samples. Cellular and substructure impacts on [^18^F]PI-2620 signal contributions were examined in depth using a PSP autoradiography sample with limited copathology. Finally, we exploited the combined acquired knowledge to create an optimized target region for the detection of cortical tau pathology in patients with 4R-tauopathies.

## Materials and methods

### Study design

In this translational study, we combined assessments of tau PET, in vitro autoradiography, quantitative tau immunohistochemistry, and cellular tracer uptake using a 4R-tauopathy mouse model and human samples consisting of patients with 4R-tauopathies and disease controls.

#### Small animal experiments

All small animal experiments were approved by the local animal care committee of the Government of Upper Bavaria (Regierung Oberbayern, approval number: ROB-55.2-2532.Vet_02-15-210, ROB-55.2-2532.Vet_02-19-26). The experiments were overseen by a veterinarian and conducted in compliance with the ARRIVE guidelines and in accordance with the U.K. Animals (Scientific Procedures) Act, 1986 and associated guidelines, EU Directive 2010/63/EU for animal experiments. The animals were housed in a temperature- and humidity-controlled environment with a 12 h light‒dark cycle and free access to food (Ssniff Spezialdiäten GmbH, Soest, Germany) and water. Anesthesia was induced before [^18^F]PI-2620 application and maintained during the PET and MR scans, with 1.5% isoflurane delivered via a mask at 3.5 L/min. All procedures were performed at the Department of Nuclear Medicine, Ludwig Maximilian University (LMU) Hospital, Munich. First, we conducted a longitudinal [^18^F]PI-2620 PET/MRI study in a 4R-tau mouse model (PS19) and age-matched wild-type mice (*n* = 10 each, all female) using regional tau PET signals and volumetric measures as endpoints. PS19 transgenic mice express mutated human microtubule-associated protein tau (MAPT) under the control of the mouse prion protein (Prnp) promoter. This transgene encompasses the P301S mutation linked to the disease and contains four microtubule-binding domains alongside an N-terminal insert (4R/1N) [57]. Next, we performed immunohistochemistry in a subset of these PS19 and wild-type mice (*n* = 4 each) to characterize the regional tau abundance and cellular contributions to tau pathology. Cell sorting after tau radiotracer injection was applied in another subset of PS19 and wild-type mice (*n* = 5 each) to determine the cellular origin of the tau PET signals.

#### Human analyses

A key experiment of the study consisted of a correlation analysis between regional [^18^F]PI-2620 tau PET signals and the abundance of fibrillary tau pathology in autopsy samples from patients with definite PSP (*n* = 6) and disease controls (*n* = 2, amyotrophic lateral sclerosis, TDP-43-positive frontotemporal lobe degeneration). In this sample, we performed a quantitative correlation analysis between tau PET signals, autoradiography, and the abundance of AT8-positive tau pathology. An additional autopsy sample from deceased patients with PSP presenting with limited copathology (*n* = 16) was used to determine the contributions of tau-positive neurons and tau-positive astrocytes to the [^18^F]PI-2620 autoradiography signals. To this end, the abundance of AT8-positive tau pathology in subfields (≥ 8) of the frontal cortex and basal ganglia sections was differentiated between neurons and astrocytes using a data-driven tissue classifier. An orienting a priori sample size calculation suggested a minimum of *n* = 6 subjects or subfields to achieve a statistical power of 0.8 at *α* = 0.05 for the detection of a meaningful explanation of variance (*β* ≥ 0.3; residual variance of 0.15) by two predictors (i.e., neuronal and astrocytic tau abundances). The tissue samples from all the autopsy cases investigated were provided by Neurobiobank Munich, LMU Munich. They were collected according to the guidelines of the local ethics committee, and the usage of the material for this project was additionally approved (application number 19-244). Finally, the combined findings were used to evaluate the emerging gray matter/white matter boundary target region for a tau PET assessment of 4R-tau pathology in the cortex of patients with PSP (*n* = 17) compared with controls (*n* = 9). All patients and controls who underwent in vivo PET imaging provided informed written consent. The study was conducted in accordance with the principles of the Declaration of Helsinki, and approval was obtained from the local ethics committee (application numbers 17-569 and 19-022).

### Small animal PET/MRI imaging

#### PET/MRI acquisition

All rodent PET procedures followed an established standardized protocol for radiochemistry, acquisition times, and postprocessing using a PET/MRI system. All the mice were scanned with a 3 T Mediso nanoScan PET/MR scanner (Mediso Ltd., Hungary) with a triple-mouse imaging chamber. Two 2-min anatomical T1 MR scans were performed prior to tracer injection (head receiver coil, matrix size 96 × 96 × 22, voxel size 0.24 × 0.24 × 0.80 mm^3^, repetition time 677 ms, echo time 28.56 ms, and flip angle 90°). The injected dose of [^18^F]PI-2620 delivered in 200 µl saline via intravenous injection was 12.7 ± 2.1 MBq. PET emission was recorded in a dynamic 0–60 min window. The frames used were 6 × 10, 2 × 30, 3 × 60, 5 × 120, 5 × 300, and 5 × 600. The list-mode data within the 400–600 keV energy window were reconstructed using a 3D iterative algorithm (Tera-Tomo 3D, Mediso Ltd., Hungary) with the following parameters: matrix size of 55 × 62 × 187 mm^3^, voxel size of 0.3 × 0.3 × 0.3 mm^3^, 8 iterations, and 6 subsets. Decay, random, and attenuation corrections were applied. The T1 image was used to create a body–air material map for attenuation correction. We longitudinally studied PS19 (*n* = 10) and age-matched wild-type mice (*n* = 10, WT; C57BL6) at 5.9, 7.7, 10.2, and 12.4 months of age. The sample size was selected based on the assumption of detecting a 10% difference between genotypes at the latest time point with a power of 0.8, applying an *α* of 0.05. No randomization was used to allocate the experimental units due to the absence of any intervention. No dropouts were registered; hence, all the mice were included in the subsequent analysis. Blinding was not applied during the scanning process, but it was implemented during image analysis, where an automatic coregistration step guaranteed reader independence [38].

#### PET/MRI analyses

The normalization of the PET data was performed by calculating the volume of distribution (V_T_) images obtained from the full dynamic scan, as described previously for different tracers [6, 56]. Briefly, we generated V_T_ images with an image-derived input function using the methodology described by Logan et al. implemented in PMOD. The plasma curve was obtained from a standardized bilateral VOI placed in the left ventricle. A maximum error of 10% and a V_T_ threshold of 0 were selected for modeling the full dynamic imaging data. Furthermore, 20–40 min static [^18^F]PI-2620 images were analyzed as a readout matching the scRadiotracing normalization. We applied the striatum as a reference tissue to decrease the variability at the individual subject level and calculated the V_T_ ratio and standardized uptake value ratio (SUVR) for images. The reference tissue was validated by analyzing V_T_ images from PS19 and WT mice, which confirmed that no differences in V_T_ in the striatum (8.4 mm^3^) were observed between genotypes. Predefined volumes of interest were delineated by spheres in the brainstem (4.2 mm^3^) and the entorhinal cortex (2.8 mm^3^), guided by regions of the Mirrione atlas but eroded to avoid the spill-in of adjacent brain structures (Supplemental Fig. 1). These target regions served for the extraction of PET values for all the mice.

The MRI volumetric analysis was performed in a blinded manner on coronal sections by manual delineation of the cerebellum, the brainstem, and the striatum (each in three adjacent planes) using PMOD (Supplemental Fig. 1). We conducted a test–retest procedure to ensure the reliability of MRI segmentation, which displayed a high congruency (*r*) of > 0.9 across 10 test cases. The cerebellum and brainstem were considered as a combined hindbrain region.

### scRadiotracing

#### Mouse brain dissociation

Five PS19 and five WT mice underwent scRadiotracing [7, 8, 56] immediately after the tau PET scan. An Adult Brain Dissociation Kit (mouse and rat) (Miltenyi Biotec, 130-107-677) was used for brain dissociation according to the manufacturer's instructions. Adult mouse brains were dissected, briefly washed with phosphate-buffered saline (PBS), cut into eight pieces, and dissociated with enzyme mixtures 1 and 2 using a gentleMACS^™^ Octo Dissociator (Miltenyi Biotec, 130-096-427). The dissociated cell suspension was applied to a prewet 100 µm cell strainer (Falcon, 352,360). The cell pellet was resuspended in cold PBS and cold debris removal solution. Cold PBS was gently overlaid on the cell suspension. The mixture was centrifuged at 4 °C and 3000×*g* for 10 min with acceleration and deceleration set at 5. The two top phases were removed entirely. The cell pellets were collected and resuspended in 1 ml of cold red blood cell removal solution, followed by 10 min of incubation. The cell pellets were collected for astrocyte and subsequent neuronal isolation via magnetic activated cell sorting (MACS) [7, 8, 56].

#### Isolation of astrocytes

An Adult Brain Dissociation Kit for mouse and rat (Miltenyi Biotec, 130-107-677) was used according to the manufacturer's instructions. The prepared cell pellets were resuspended in 80 µl of AstroMACS separation buffer (Miltenyi Biotec, 130-117-336) per 10^7^ total cells. Then, 10 μL of FcR blocking reagent was added, and the mixture was incubated for 10 min in the dark at 4 °C. Next, 10 μL of Anti-ACSA-2 MicroBeads was added, and the mixture was incubated for 15 min in the dark at 4 °C. The cells were washed by adding 1 mL of AstroMACS separation buffer and centrifuged at 300×*g* for 5 min. The cell pellets were resuspended in 500 μL of AstroMACS separation buffer. The prewet MS columns (Miltenyi Biotec, 130-042-201) were placed in an OctoMACS Separator (Miltenyi Biotec, 130-042-109). The cell suspensions were applied onto the column, followed by washes with 3 × 500 µL of AstroMACS separation buffer. The flow-through was collected and contained nonastrocytic cells as an astrocyte-depleted fraction. The columns were removed from the magnetic field, and the astrocytes were flushed out using 3 ml of AstroMACS separation buffer.

#### Isolation of neurons

A Neuron Isolation Kit, mouse (Miltenyi Biotec, 130-115-390) was used as previously reported [20], according to the manufacturer's instructions. The astrocyte-depleted cell pellets were resuspended in 80 µl of PBS–0.5% bovine serum albumin (BSA) buffer per 10^7^ total cells. Twenty microliters of nonneuronal cell biotin–antibody cocktail was added, and the mixture was incubated for 5 min in the dark at 4 °C. The cells were washed and centrifuged at 300×*g* for 5 min. The cell pellets were again resuspended in 80 μL of PBS–0.5% BSA buffer per 10^7^ total cells. Next, 20 μL of anti-biotin microbeads was added, and the mixture was incubated for 10 min in the dark at 4 °C. The volume was adjusted to 500 µl per 10^7^ total cells with PBS–0.5% BSA buffer, and then, magnetic separation was performed. The prewet LS columns (Miltenyi Biotec, 130-042-401) were placed in a QuadroMACS^™^ Separator (Miltenyi Biotec, 130-090-976). The cell suspensions were applied onto the columns. The columns were washed with 2 × 1 ml of PBS–0.5% BSA buffer. The flow-throughs containing the unlabeled cells were collected as the neuron-enriched fractions. The columns were removed from the magnetic field, and the nonneuronal cells were flushed out with 3 ml of PBS–0.5% BSA buffer [56].

#### Gamma emission, flow cytometry, and calculation of single-cell tracer uptake

The radioactivity concentrations of the cell pellets were measured with a highly sensitive gamma counter (Hidex AMG Automatic Gamma Counter, Mainz, Germany) are reported relative to the activity in the whole brain, with decay correction to the time of tracer injection for the final activity calculations.

Flow cytometry staining was performed at 4 °C. After the gamma emission measurement, the cell suspension was centrifuged at 400×*g* for 5 min, and the supernatant was aspirated completely. The cell pellet was then resuspended in 100 µl of cold D-PBS containing fluorochrome-conjugated antibodies recognizing mouse CD11b and ACSA-2 (Miltenyi Biotec, 130-113-810 and 130-116-247) at a 1:100 dilution and incubated for 10 min at 4 °C in the dark. The samples were washed with 2 ml of D-PBS and centrifuged for 5 min at 400×*g*. Finally, the cell pellets were resuspended in 500 μl of D-PBS, and the samples were immediately used for flow cytometry with an MACSQuant® Analyzer as a quality control for MACS. Absolute cell numbers were acquired for all the samples. The purity of the astrocyte-enriched cell pellet was determined via ACSA-2 staining. For the assessment of purity within the neuron-enriched fraction, the proportion of remaining CD11b- and ACSA-2-positive cells was determined and subtracted from the total number of cells within the pellet. The CD11b-/ACSA-2-negative fraction was considered neurons and validated using CD90.1 as a neuronal marker for C57BL6 mice (consistently > 85%).

The measured radioactivity (Bq) of the cell pellets was divided by the specific cell number in the pellet, resulting in the calculated radioactivity per cell. The sufficient sensitivity of a single readout was determined as a ≥ twofold ratio between the cell pellet radioactivity and the background measurement, with a total procedure duration of 6–7 h from tracer injection to radioactivity measurement in the enriched cell pellets. The radioactivity per cell was normalized to the injected radioactivity and body weight (%ID*BW).

### Small animal immunohistochemistry

An additional number of four female PS19 mice and four wild-type mice were used for immunohistochemistry, as the same brains could not be used for both scRadiotracing analysis and immunohistochemical staining. Fifty-micron-thick slices were cut in the sagittal plane using a vibratome (VT1200S, Leica Biosystems). Slices were treated with blocking solution (10% normal goat serum and 10% donkey serum in 0.3% Triton and PBS to a total volume of at least 200 μl per well/slice) for 3 h at RT. The following primary antibodies were used: chicken anti-GFAP (1:500; ab5541; Merck Millipore, Darmstadt, Germany), mouse anti-AT8 (1:1000; ab5541; Merck Millipore, Darmstadt, Germany), and rabbit anti-MAP2 (1:500) diluted in blocking solution (5% normal goat serum and 5% donkey serum in 0.3% Triton and PBS to a total volume of at least 200 μl per well/slice). The antibodies were applied to the slices and subsequently incubated for ~ 48 h at 4 °C on a horizontal shaker. The following secondary antibodies were used: goat anti-rabbit Alexa Fluor 488 (1:500), goat anti-chicken Alexa Fluor 555 (1:500), and goat anti-mouse Alexa Fluor 647 (1:500) diluted in PBS. Slices were incubated for 2–3 h at RT on a horizontal shaker in the dark. After 3 × 10 min washes with PBS, the slices were mounted and cover slipped with fluorescence mounting medium containing DAPI (Dako, Santa Clara, USA).

Three-dimensional images were acquired with an Apotome microscope (Zeiss Oberkochen, Germany) using 10 × and 40 × objectives. The analysis programs Zeiss blue and ImageJ were used for quantification. Z-stack images (10 μm) were acquired with a 10 × objective. Each signal (AT8 and GFAP) was quantified as the % area of the entire scanning frame.

Single optical sections were acquired using a 40 × objective, and the AT8 signal was analyzed, because the perceptual signals were MAP2- and GFAP-positive. To this end, we created a mask of the AT8 signal and transferred it into GFAP-positive astrocytes and MAP2-positive neuronal structures. After local brightness/contrast adjustments and background subtraction, we set a fixed threshold and calculated the AT8 area (%) inside the mask of the GFAP-positive astrocytes and MAP-positive neurons.

### Human postmortem samples

#### Human samples

For PET of autopsy samples, we included all patients who underwent [^18^F]PI-2620 tau PET prior to death, donated their brain to the Munich Brain Bank, and underwent a tissue workup by 31 March 2024 (*n* = 8; Supplemental Table 1). The formalin-fixed and paraffin-embedded tissue blocks of one hemisphere were used for AT8 and autoradiography analyses. The tissue from the medial frontal gyrus and basal ganglia, including the globus pallidus, was available for seven patients with definite PSP, one patient with FTLD-TDP, and one patient with FTLD/MND-TDP. For in-depth analyses of the origins of the cellular and structural radiotracer signals, we selected samples with limited α-synuclein, TDP-43, and FUS pathology from the Munich Brain Bank. Limited β-amyloid pathology was tolerated, resulting in a total sample size of *n* = 16 (Supplemental Table 2). Intact medial frontal gyrus tissue was available for fourteen patients, and intact basal ganglia tissue, including the globus pallidus, was available for seven patients. We conducted [^18^F]PI-2620 autoradiography and AT8 immunohistochemistry on postmortem brain tissues from *n* = 4 patients with clinically diagnosed Parkinson’s disease (PD) to examine the specificity of tracer binding (Supplemental Table 3).

#### Immunohistochemistry

Immunohistochemistry was performed on 4 µm-thick sections of formalin-fixed and paraffin-embedded tissue using standard techniques. Immunohistochemical tau staining was performed semiautomatically using a BenchMark device (Ventana, now Hoffmann-LaRoche, Basel, Switzerland) with a mouse monoclonal AT8 antibody raised against hyperphosphorylated tau (Ser202/Thr205, 1:200, Invitrogen/Thermo Fisher, Carlsbad, CA, USA), as well as with the mouse monoclonal isoform-specific tau antibodies RD3 (8E6/C11) and RD4 (1E1/A6), on adjacent sections of those used for ARG. The immunostained sections were digitized at 20 × magnification with a Mirax Midi scanner (Zeiss, Carl Zeiss MicroImaging GmbH, Jena, Germany). For frontal cortex (medial frontal gyrus) and globus pallidus analyses, 8–12 regions of interest (subfields) were drawn manually per section, and the AT8-positive tau load (%) was quantified using ZEN 3.4 blue edition software (Zeiss, Jena, Germany).

Similar to previous approaches, i.e., by Rittman et al. [40], we aimed to subdivide the AT8-positive tau load into different underlying cell types. Therefore, we used semiautomated object characterization and recognition to differentiate neurofibrillary tangles (NFTs), coiled bodies (CBs), and tufted astrocytes (TAs) based on several parameters of morphological characteristics. Blinding was ensured by randomizing and renaming the digital images, effectively eliminating any potential bias associated with the sample origin. Single NFT, CB, and TA (*n* = 15–20 objects per slice) were manually selected to define object thresholds, including the size (area), diameter, ellipse axis, perimeter, intensity, grade of circularity, roundness, and compactness. Specific masks were generated for positive NFTs, CBs, and TAs and were uniformly applied to all sections (Supplemental Fig. 2). This combination of blinding, randomization, and consistent mask application ensured reproducibility and allowed for the precise quantification of AT8 immunoreactivity across the entire sample set. Due to substantial overlap of object characteristics, we subsequently defined NFT and CB as a combined group of AT8-positive cells with high density. Notably, tau fragments (TFs) were partially included in the TA channel, resulting in two analysis channels (NFT/CB and TA/TF). This finding was substantiated by the correlation analysis between AT8 positivity and autoradiography signals in single subjects, which revealed similar associations of neuron- and oligodendrocyte-enriched regions with autoradiography signals. The final segmentation resulted in NFT/CB AT8-area-%, TA/TF AT8-area% and intensities within 8–12 subfields per analyzed section.

For the correlation analysis between in vivo PET imaging data and the tau load at autopsy, composite regions of interest in the medial frontal gyrus and in the globus pallidus (internal and external parts) were used.

#### Autoradiography

For direct comparison with the autoradiography signal, tau immunostaining of formalin-fixed and paraffin-embedded tissue blocks from 16 PSP patients and 4 PD patients and three brain regions (frontal cortex, putamen, and pallidum) was performed. For each patient and brain region, autoradiography with [^18^F]PI-2620 was performed on ≥ 4 sections, as described previously [55]. Briefly, the sections were incubated for 45 min (21.6 μCi/ml after dilution to a volume of 50 ml with phosphate-buffered saline solution, pH 7.4, specific activity of 480 ± 90 GBq/μmol), washed, dried, placed on imaging plates for 12 h, and scanned at 25.0 µm resolution. Regions of interest were drawn on each sample using the AT8 staining of the adjacent section, thus serving to anatomically define subfields in the frontal cortex (gray matter and white matter). An AT8-negative region in the white matter was used as the reference region, and the ratios between the subfield target regions and the reference region were calculated. Each subfield region was labeled with a cortical or gray matter/white matter boundary. Binding ratios were correlated with a semiquantitative AT8 assessment using Pearson’s correlation coefficient after testing for normality and subjected to a regression analysis (neuronal vs. astrocytic tau).

### Human PET imaging and analysis

#### Tau PET image acquisition and preprocessing

[^18^F]PI-2620 was synthesized as previously described [47]. The injected dose ranged between 156 and 223 MBq and was applied as a slow (10 s) intravenous bolus injection. Positron emission tomography (PET) imaging was performed in a fully dynamic setting (scan duration: 0–60 min postinjection) using a Siemens Biograph True point 64 PET/CT system or a Siemens mCT system (Siemens, Erlangen, Germany). The dynamic brain PET data were acquired in three-dimensional list mode over 60 min and reconstructed into a 336 × 336 × 109 matrix (voxel size: 1.02 × 1.02 × 2.03 mm^3^) using the built-in ordered subset expectation maximization (OSEM) algorithm with 4 iterations, 21 subsets, and a 5 mm Gaussian filter on the Siemens Biograph and with 5 iterations, 24 subsets, and a 5 mm Gaussian filter on the Siemens mCT. Low-dose CT served for attenuation correction. Frame binning was standardized to 12 × 5 s, 6 × 10 s, 3 × 20 s, 7 × 60 s, 4 × 300 s, and 3 × 600 s. Image-derived input functions were generated by manual and automated extraction of the PET standardized uptake value (SUV) from the carotid artery over a 60-min dynamic PET scan.

Via manual extraction, the blood activity concentration in the bilateral carotid artery was detected in early frames of the dynamic PET images, and spheres with a diameter of 5.0 mm were placed as volumes of interest (VOIs) in the pars cervicalis of the internal carotid artery prior to entering the pars petrosal using PMOD version 4.2 (PMOD Technologies, Zürich, Switzerland). The activity concentration over time was calculated from the average values of the VOI.

#### Tau PET quantification

Volume distribution (VT) images were calculated with the IDIFs using Logan plots [30], which assume that the data become linear after an equilibration time t*. t* was fitted based on the maximum error criterion, which indicates the maximum relative error between the linear regression and the Logan-transformed measurements in the segment starting from t*. The maximum error was set to 10%. The percentage of masked pixels was set to 0%. The putamen, which was defined by manual placement of a VOI (sphere with a diameter of 10 mm), served as the tissue region.

All VT images were transformed to MNI space via 20–40 min coregistration using the established [^18^F]PI-2620 PET template [18]. The automated brain normalization settings in PMOD included nonlinear warping, 8 mm input smoothing, equal modality, 16 iterations, a frequency cutoff of 3, regularization of 1.0, and no thresholding.

For the PET-to-autopsy correlation analysis, VT ratios (temporal white matter reference region [10]) were obtained in the medial frontal gyrus and in the globus pallidus as PSP target regions (see immunohistochemistry), which were predefined by the atlas of the basal ganglia [25], the Brainnetome atlas [17], and the Hammers atlas [21]. The rationale was to employ a matched quantification strategy between PET and autoradiography.

#### GM/WM target region

All patients and controls used for this dedicated analysis underwent T1-weighted structural MRI on a 3 T Siemens Magnetom PRISMA or SKYRA Scanner and [^18^F]PI-2620 tau PET in a fully dynamic setting (0–60 min postinjection) using pre-established standard PET scanning parameters [46]. Our in-house [^18^F]PI-2620 synthesis and quality control pipeline has been described in detail previously [12, 19]. Dynamic [^18^F]PI-2620 tau PET images were acquired on a Siemens Biograph True point 64 PET/CT (Siemens, Erlangen, Germany) or a Siemens mCT scanner (Siemens, Erlangen, Germany) in 3D list mode over 60 min together with low-dose CT for attenuation correction. For dynamic PET image acquisition, we reconstructed late-phase tau PET images at 20–40 min p.i., which were summarized into a single frame after motion correction [9, 24, 46, 53].

All images were screened for artifacts before preprocessing. T1-weighted structural MR scans were bias-corrected and segmented into tissue types using the CAT12 toolbox (https://neuro-jena.github.io/cat12-help/). PET images were linearly coregistered to the corresponding T1 MRI data, and the intensity was normalized using a pre-established inferior cerebellar reference region [19]. Using T1 MRI data, surface reconstruction was performed using the CAT12-based cortical thickness pipeline. Surface reconstruction was performed for the GM/WM boundary and then systematically shifted to the underlying white matter, as well as to the GM/CSF boundary. Using these systematically shifted surfaces, we extracted PI-2620 tau PET SUVRs for 200 regions of the cortical Schaefer atlas to systematically determine the tau PET signal from the GM/CSF toward the GM/WM boundary and below.

### Statistics

GraphPad Prism (V10, GraphPad Software, US) and SPSS (V27, IBM, US) were used for the statistical analyses.

#### Mouse PET/MRI

Mixed linear models (Graph Pad Prism) were used to test for age × genotype effects on PS19 and WT mice, including tau PET binding and MRI volumes, between 6 and 12 months of age as indices of interest. Pearson’s correlation coefficient was calculated between brainstem tau PET binding and the brainstem volume at all investigated time points (separately for PS19 and WT mice). As a supporting analysis, we correlated tau PET binding and immunohistochemistry in the frontal cortex (*n* = 4) and the hippocampus (*n* = 3), which are brain regions with well-documented tau protein immunoreactivity [3] and limited spill-over from adjacent tissue, to ensure reliable values at the single-mouse-level of individual PS19 mice. One hippocampal section was excluded due to limited quality of the tissue material. A sample size calculation was performed (G*Power) to determine the minimal age of detectability of tau PET binding and MRI volume with cohorts of *n* = 12 PS19 mice and *n* = 12 WT mice (power = 0.8, *α* = 0.05; Supplemental Fig. 3). No corrections for multiple comparisons were applied in the small animal PET experiments, as only a limited number of target regions were selected. This approach resulted in a low Type I error rate (*α*), with moderate sample sizes, thereby adhering to the 3Rs (Reduction) principles of animal welfare.

#### Mouse immunohistochemistry

Regional coverage of AT8 and GFAP staining was compared between PS19 and WT mice with an unpaired Student’s t test. The colocalization of AT8 with neurons (MAP2 +) and astrocytes (GFAP +) was compared using an unpaired Student’s *t* test.

#### Mouse scRadiotracing

Radiotracer uptake per neuron and astrocyte was compared between PS19 and WT mice using an unpaired Student’s *t* test. Furthermore, in PS19 and WT mice, radiotracer uptake was compared between neurons and astrocytes. Pearson’s correlation coefficients were calculated between the radioactivity per cell and the tau PET signals for the combined data from the PS19 and WT mice, as well as for the subset of PS19 mice. For the whole-brain voxelwise correlation analysis between cellular tracer uptake (*n* = 5 PS19, *n* = 5 WT), statistical parametric mapping (SPM) was performed using SPM12 routines (Wellcome Department of Cognitive Neurology, London, UK) implemented in MATLAB (version 2016). Individual SUVR images were subjected to linear regression analysis with cellular tracer uptake in neurons or astrocytes (%ID*BW) as a vector in the pooled cohort of PS19 and WT mice (threshold: *p* < 0.005 uncorrected, *k* > 20 voxels). Increases in the tau PET signals in each of the five PS19 mice that underwent scRadiotracing were compared with the average tau PET signal in the WT mice. The average tau PET signal in the WT mice was considered an unspecific background signal, as the WT mice did not show any tau accumulation in the brain. As PET signals need to be recognized as a product of cellular tracer uptake and cell type abundance in the brain, we used extrapolation to estimate the total contributions of neurons and astrocytes to increases in tau-related PET signals in PS19 mice. We multiplied the individual cellular tracer uptake (astrocytes and neurons) of each mouse with published cell numbers (i.e., 71*10e^6^ neurons and 21*10e^6^ astrocytes) as a surrogate for the cell type abundance in the brains of PS19 mice [22]

$$\begin{gathered} 71{*}10{\text{e}}^{6} {*}\left( {\frac{{{\text{Bq}}}}{{{\text{neuron}}}}Mouse\# 1 + \frac{{{\text{Bq}}}}{{{\text{neuron}}}}Mouse\# 2 + \frac{{{\text{Bq}}}}{{{\text{neuron}}}}Mouse\# 3 + \frac{{{\text{Bq}}}}{{{\text{neuron}}}}Mouse\# 4 + \frac{{{\text{Bq}}}}{{{\text{neuron}}}}Mouse\# 5} \right) \hfill \\ + 21{*}10{\text{e}}^{6} {*}\left( {\frac{{{\text{Bq}}}}{{{\text{astrocyte}}}}Mouse\# 1 + \frac{{{\text{Bq}}}}{{{\text{astrocyte}}}}Mouse\# 2 + \frac{{{\text{Bq}}}}{{{\text{astrocyte}}}}Mouse\# 3 + \frac{{{\text{Bq}}}}{{{\text{astrocyte}}}}Mouse\# 4 + \frac{{{\text{Bq}}}}{{{\text{astrocyte}}}}Mouse\# 5} \right) \hfill \\ = {\text{Bq}}_{{\text{tau - PET}}} \left( {Mouse\# 1 - AVG_{WT} } \right) + {\text{Bq}}_{{\text{tau - PET}}} \left( {Mouse\# 2 - AVG_{WT} } \right) \ldots + {\text{Bq}}_{{\text{tau - PET}}} \left( {Mouse\# 5 - AVG_{WT} } \right). \hfill \\ \end{gathered}$$

We used the sum of all 5 mice for comparison instead of single mice to minimize confounding factors caused by, e.g., distinct cell type abundances in the brains of individual mice compared with those reported in the literature or the methodological variance of scRadiotracing. A paired *t* test was used to compare PET radioactivity and the extrapolated radioactivity of single cells in the cohort of five PS19 mice.

#### Human PET-to-autopsy correlation

Partial correlation coefficients were calculated for the multimodal correlation between tau abundance via immunohistochemistry, autoradiography ratios, and tau PET signals, accounting the globus pallidus and frontal cortex as cofactors across samples.

#### Human autoradiography

Neuronal and astroglial tau abundances were compared with paired *t* tests. The Kolmogorov‒Smirnov test confirmed the normality of the residuals. Neuronal and astroglial tau abundances in subfields of the frontal cortex were correlated with autoradiography binding ratios in corresponding regions (total *n*  =  129; Supplemental Fig. 4). Additionally, a linear regression analysis was performed with neuronal and astroglial tau abundances as predictors and autoradiography binding ratios as the outcome variable. At the individual level, the correlation between neuronal and astroglial tau abundances and autoradiography binding ratios was determined in 8–12 subfields per subject. These individual correlations were analyzed as a function of overall tau abundance (separately for neurons and astroglia). In basal ganglia regions, the AT8 signal intensity and AT8 occupancy were correlated with autoradiography binding ratios.

#### Human PET target region

Differences in the SUVRs of *n* = 5 different layers (GM/CSF boundary, GM toward CSF, GM toward WM, GM/WM boundary, and below the GM/WM boundary) were compared using a repeated-measures ANOVA, accounting for within-subject variability. Post hoc pairwise comparisons were performed using Tukey-adjusted estimated marginal means to identify specific differences between layers.

## Results

### [^18^F]PI-2620 tau PET monitoring reveals age-dependent increases in tracer binding in AT8-positive brain regions in the PS19 4-repeat tau mouse model

**Fig. 1:**
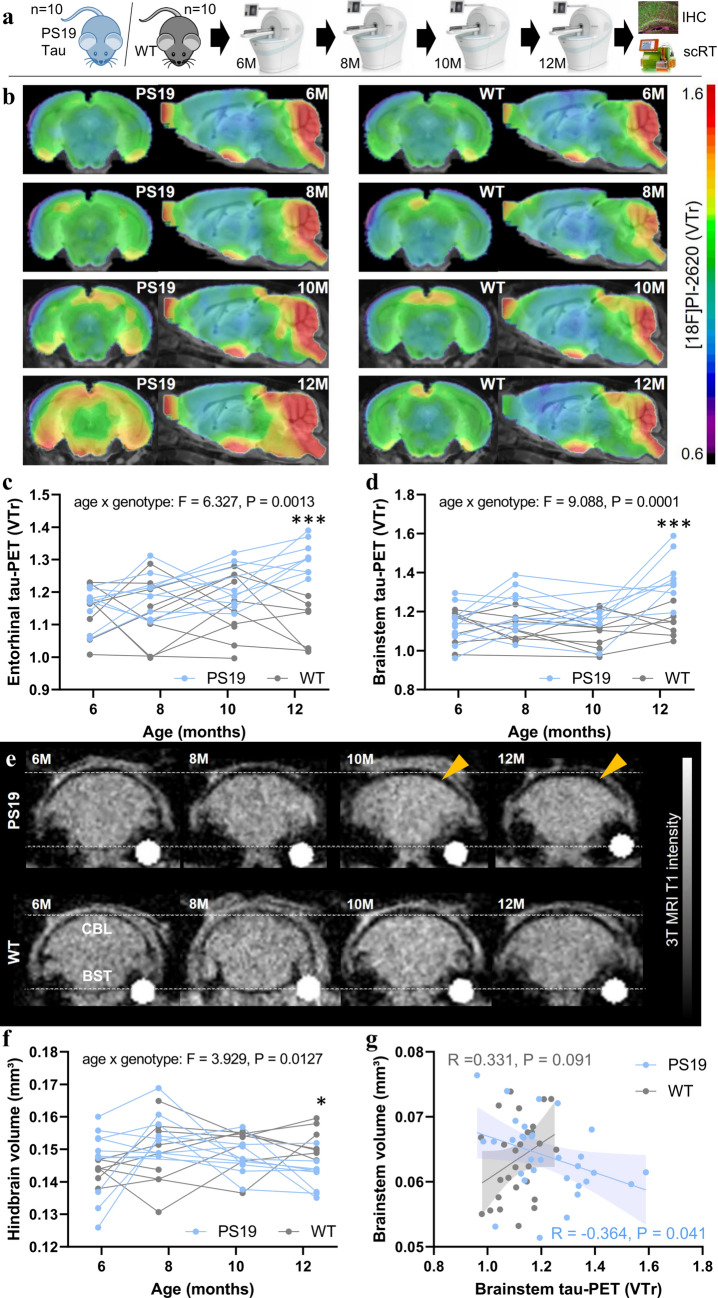
Monitoring of tau pathology and atrophy in PS19 and wild-type mice using [^18^F]PI-2620 PET/MRI. **a** Experimental workflow of serial PET/MRI imaging sessions and terminal immunohistochemistry (IHC) and single-cell Radiotracing (scRT) in PS19 and wild-type (WT) mice. **b** Coronal and axial group average [^18^F]PI-2620 PET images obtained using an MRI template show the monitoring of radiotracer binding (volume of distribution ratios, VT; striatal reference), with pronounced temporal and brainstem patterns in aged PS19 mice (*n* = 8–10) compared with WT mice (*n* = 8–10). **c**, **d** Mixed linear models of entorhinal and brainstem [^18^F]PI-2620 PET signals indicate a significant age × genotype effect and elevated tau PET signals in PS19 mice compared with WT mice at 12 months of age. **e** Examples of serial MRI atrophy patterns in an individual PS19 mouse compared with a WT mouse. Coronal slices are shown with indications of the cerebellum (CBL) and brainstem (BST) regions. The orange arrows highlight atrophy in PS19 mice. **f** Mixed linear model of hindbrain volume indicating a significant age × genotype effect and a decreased hindbrain volume in PS19 mice compared with WT mice at 12 months of age. **g** Association between tau PET signals and brain volume in the brainstem of PS19 and WT mice across all investigated time points showing greater atrophy in the presence of high tau PET signals, specifically in PS19 mice

First, we investigated whether [^18^F]PI-2620 tau PET has sufficient sensitivity to detect an in vivo tracer signal in PS19 mice, which accumulate 4R-tau pathology, compared with that in wild-type (WT) mice (Fig. 1a). Longitudinal tau PET scans from 6 to 12 months of age revealed an increasing PET signal and a significant genotype × age interaction effect on the entorhinal cortex (*F*_(3,40)_ = 6.33, *p* = 0.0013) and brainstem (*F*_(3,40)_ = 9.09, *p* = 0.0001) of PS19 mice compared with those of WT mice (Fig. 1b–d). The cerebellum did not qualify as a suitable target region in mice because of the strong spill-over of the adjacent skull relative to the brain (Fig. 1b). Compared with WT mice, late-stage PS19 mice presented strongly elevated [^18^F]PI-2620 tau PET signals in the entorhinal cortex (+ 19%, Cohen’s *d* = 3.26, *p* = 0.0003) and brainstem (+ 21%, Cohen’s *d* = 2.47, *p* = 0.0009). Power calculations with standardized sample sizes of *n* = 12 mice per genotype indicated an age of 9.7 months for the earliest detection of signal alterations in cohorts of PS19 mice compared with WT mice at a predefined power of 0.8 (Supplemental Fig. 3). Individual PET images of PS19 and WT mice at 12 months of age are shown in Supplemental Fig. 5. Regional AT8 staining correlated with regional tau PET signal enhancement at the final time point (*R* = 0.921, *p* = 0.0032; Supplemental Fig. 6). The volumetric 3 T MRI analysis revealed a decrease in the hindbrain volume of late-stage PS19 mice compared with WT mice (− 7%, Cohen’s *d* = 1.98, *p* = 0.0062) and a weaker genotype × age interaction effect (*F*_(3,59)_ = 3.93, *p* = 0.013) than tau PET (Fig. 1e, f). The earliest detection of differences in hindbrain volume was estimated at 11.7 months of age (Supplemental Fig. 3). PS19 mice with high tau PET signals in the brainstem were characterized by a substantial volume loss in the brainstem, whereas this association was not present in WT mice (Fig. 1g).

### Immunohistochemistry shows the dominance of neuronal tau over astrocytic tau in PS19 mice

**Fig. 2:**
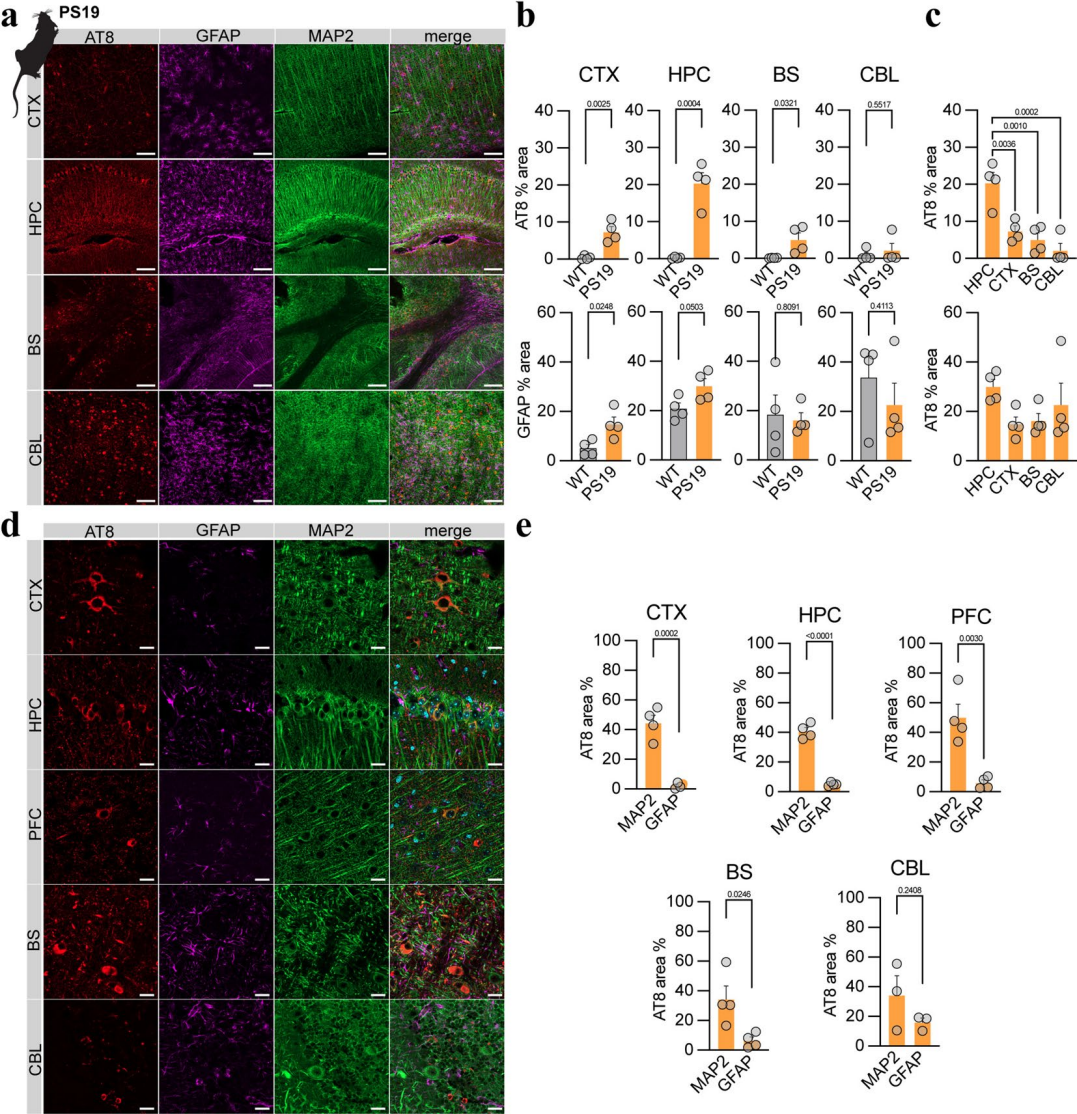
Immunohistochemical assessments of tau pathology, reactive astrocytes, and neurons in PS19 mice. **a** Overview of sections from PS19 mice showing pTau (AT8, red), astrocytes (GFAP, purple), and neurons (MAP2, green), as well as merged images of the cortex (CTX, motor cortex, and somatosensory cortex), hippocampus (HPC), cerebellum (CBL), and brainstem (BS). Scale bar = 100 µm. **b** Quantitative comparison of AT8 occupancy in target regions between wild-type (WT) and PS19 mice, as well as a comparison of PS19 AT8 occupancy across target regions. **c** Quantitative comparison of GFAP occupancy in target regions between wild-type (WT) and PS19 mice, as well as a comparison of PS19 GFAP occupancy across target regions. **d** High-magnification images of sections from PS19 mice showing pTau (AT8, red), astrocytes (GFAP, purple), and neurons (MAP2, green), as well as merged images of the cortex (CTX), hippocampus (HPC), cerebellum (CBL), and brainstem (BS). Scale bar = 20 µm. **e** Quantitative comparison of AT8 occupancy between neurons (MAP2-positive) and astrocytes (GFAP-positive) in target regions of PS19 mice. *CTX* cortex, *HIP* hippocampus, *PFC* prefrontal cortex, *BS* brainstem, *CBL* cerebellum

Next, we assessed the detailed regional sources of tau pathology in the brains of PS19 mice using immunohistochemistry. Consistent with previous work, a greater abundance of AT8-positive tau pathology was observed in the hippocampus (+ 20.1%, *p* = 0.0004), cortex (+ 7.0%, *p* = 0.0025) and brainstem (+ 5.0%, *p* = 0.0321) of PS19 mice than in those of age-matched WT mice at 12 months (Fig. 2a, b) [57]. In contrast, the cerebellum showed no significant increase in the AT8-positive area (1.3%, *p* = 0.552). Similarly, stronger GFAP reactivity, a surrogate for reactive gliosis, was observed in the frontal cortex (+ 9.7%, *p* = 0.025) and the hippocampus (+ 9.2%, *p* = 0.050; Fig. 2a, c) of PS19 mice than in those of WT mice [41].

The colabeling of AT8 with GFAP to indicate astroglia and MAP2 to mark neuronal soma, including somatodendritic structures, revealed that tau aggregation predominantly occurred in neurons (somata and putatively postsynaptic compartments), with a significantly lower level observed in astrocytes (2–18-fold, all target regions *p* < 0.05; Fig. 2e). Collectively, these data reflect signals obtained from PS19 mice subjected to tau PET imaging and depict the predominant neuronal origin of tau pathology.

### Increased neuronal [^18^F]PI-2620 uptake in late-stage PS19 mice translates to an in vivo PET signal

**Fig. 3:**
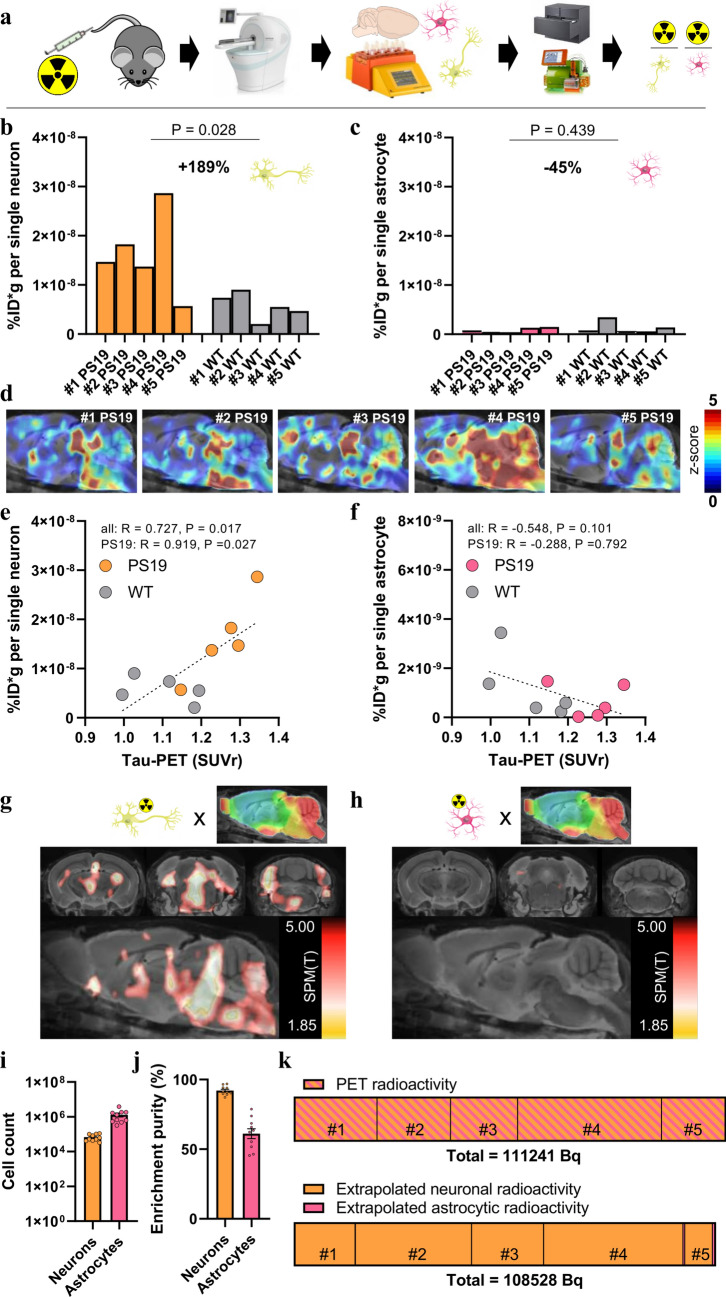
Cell sorting after radiotracer injection identifies neurons as the predominant origin of [^18^F]PI-2620 tau PET signals. **a** Experimental workflow of PET/MRI with subsequent cell sorting of neurons and astrocytes prior to the determination of radioactivity per isolated cell via gamma emission measurements and flow cytometry. **b**, **c** Comparison of radioactivity per isolated neuron and astrocyte between PS19 (*n* = 5) and wild-type (WT, *n* = 5) mice. Each bar represents an individual animal. **d** Sagittal sections obtained via an MRI template showing z score images (vs. WT) of all investigated PS19 mice (*n* = 5). Each image represents an individual animal. **e**, **f** Quantitative correlation between brainstem tau PET signals and radioactivity per single neuron or astrocyte. Pearson’s correlation coefficients are provided for the combined data from PS19 and WT mice (regression line with 95% confidence interval), as well as for the subset of PS19 mice. **g**, **h** Data-driven voxelwise correlation between radioactivity per single neuron or astrocyte and [^18^F]PI-2620 tau PET images using statistical parametric mapping of the combined sample of PS19 and WT mice. Radiotracer uptake per neuron correlated with the distribution pattern of tau pathology in PS19 mice, whereas radiotracer uptake per astrocyte did not correlate with tau PET patterns. **i**, **j** Cell count and purity of isolated cells (neurons and astrocytes) as benchmark indices of the cell sorting procedure. **k** The increase in PET radioactivity in PS19 mice (*n* = 5) compared with that in WT mice matches the increase in radioactivity determined by the number of isolated cells. The radioactivity per single cell was extrapolated from the established cell numbers in the mouse brain (71 × 10e^6^ neurons, 21 × 10e^6^ astrocytes)

We performed scRadiotracing in a subset of five transgenic and five WT mice immediately after the final tau PET session to determine the cellular source of [^18^F]PI-2620 binding in PS19 mice (Fig. 3a). Neurons and astrocytes were isolated using MACS, and radioactivity was measured in enriched cell pellets. Strikingly, we observed a 1.89-fold higher [^18^F]PI-2620 uptake per single neuron in PS19 mice than in the single neurons of WT mice (*p* = 0.028; Fig. 3b). In contrast, [^18^F]PI-2620 uptake per single astrocyte did not differ between PS19 and WT mice (*p* = 0.439; Fig. 3c). In PS19 mice, neuronal tracer uptake was 27-fold higher than that in astrocytes (*p* = 0.023), whereas in WT mice, [^18^F]PI-2620 uptake by neurons was higher than that by astrocytes (fivefold, *p* = 0.009). Individual tau PET z score maps of PS19 mice matched the magnitude of single-cell [^18^F]PI-2620 uptake in neurons (Fig. 3d). Furthermore, late static tau PET quantification in a predefined hindbrain region was correlated with individual neuronal tracer uptake in the whole sample (*R* = 0.727, *p* = 0.017) and in PS19 mice (*R* = 0.919, *p* = 0.027; Fig. 3e), whereas no significant associations were observed between tau PET signals and individual astrocytic tracer uptake (Fig. 3f). We correlated individual neuronal and astrocytic tracer uptake with voxelwise tau PET signals using SPM to increase regional flexibility. Neuronal [^18^F]PI-2620 uptake was correlated with regional PET signals in the brainstem, midbrain, and entorhinal cortex (Fig. 3g), whereas astrocytic [^18^F]PI-2620 uptake was not significantly correlated with regional tau PET signals (Fig. 3h). Flow cytometry confirmed the sufficient yield (Fig. 3i) and purity (Fig. 3j) of neurons and astrocytes. Thus, we questioned whether the magnitude of cell-specific tracer uptake in individual PS19 animals corresponds to the alterations observed in PET signals. Considering 71*10e^6^ neurons and 21*10e^6^ astrocytes in the rodent brain, we found that cellular radioactivity (7.7–36.5 kBq per animal) explained the specific increase in the tau PET signal (16.4–37.2 kBq per animal; *p* = 0.880), which also closely matched the summations of cellular and PET-related radioactivity measures across the five PS19 mice studied (Fig. 3k).

### [^18^F]PI-2620 tau PET signals correlate strongly with regional tau abundance in deceased patients with PSP and disease controls

**Fig. 4:**
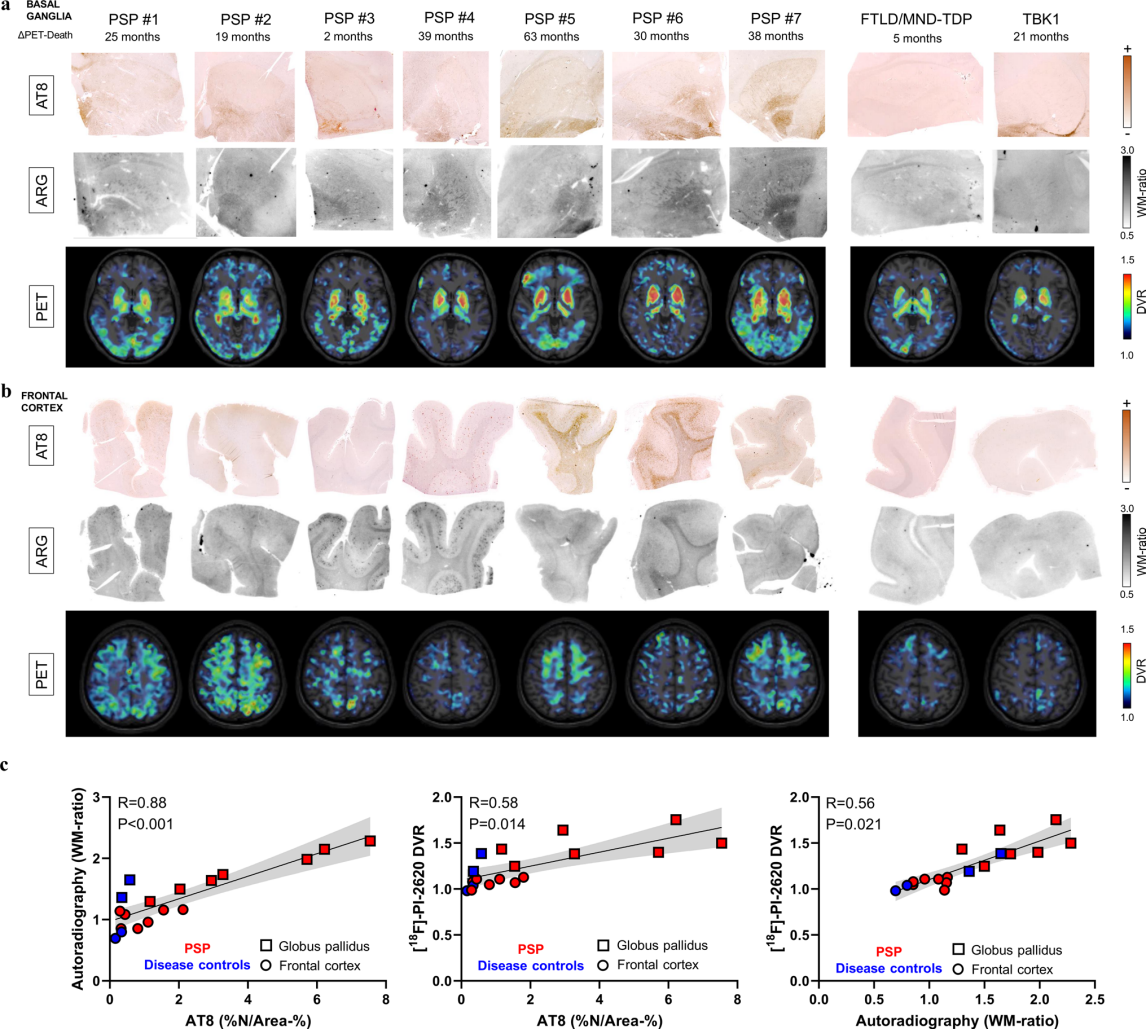
Correlations of in vivo PET signals with tau abundance and autoradiography signals in autopsy samples. **a** Basal ganglia AT8 immunohistochemistry together with [^18^F]PI-2620 autoradiography of adjacent sections and axial [^18^F]PI-2620 tau PET signals in basal ganglia sections prior to death. Images are shown for all seven investigated patients with definite PSP and two patients with TAR DNA-binding protein 43 (TDP-43)-positive frontotemporal lobar degeneration (FTLD-TDP): one with FTLD/MND-TDP and one with FTLD-TDP related to a TANK-binding kinase 1 (TBK1) mutation. **b** Frontal medial gyrus AT8 immunohistochemistry together with [^18^F]PI-2620 autoradiography of adjacent sections and axial [^18^F]PI-2620 tau PET signals in cortical sections prior to death. Images are displayed for seven patients with definite PSP and two disease controls, as indicated in (A). **c** Multimodal quantitative partial correlation between tau abundance, autoradiography signals and tau PET signals. Linear regression lines (including 95% confidence intervals) were calculated, taking into account the globus pallidus and frontal medial gyrus as cofactors, in samples derived from seven patients with definite PSP, one patient with FTLD/MND-TDP, and one patient with FTLD related to a TBK1 mutation. R indicates Pearson’s correlation coefficient. %N/Area-% = AT8 occupancy of neurofibrillary tangles and coiled bodies

Next, we examined whether in vivo signals of the tau PET tracer [^18^F]PI-2620 are determined by tau neuropathology. To this end, we investigated a small cohort of nine patients who underwent [^18^F]PI-2620 PET in vivo, with subsequent donation of their brains for autopsy. Seven patients were classified as having definite PSP, and two patients were classified as having TAR DNA-binding protein 43 (TDP-43)-positive frontotemporal lobar degeneration (FTLD-TDP): one with FTLD/MND-TDP and one with FTLD-TDP related to a TANK-binding kinase 1 (TBK1) mutation (Supplemental Table 1). The globus pallidus showed greater visual and quantitative AT8 occupancy than did the medial frontal gyrus in patients with definite PSP, which was well reflected by the corresponding autoradiography signals (Fig. 4a, b). Tau PET, autoradiography, and AT8 immunohistochemistry indicated higher signals or occupancy in patients with definite PSP than in disease controls (Fig. 4a, b). Notably, the TBK1 mutation carrier presented mild AT8-positive tau copathology in the globus pallidus and in the putamen and moderate AT8-Co-pathology in the nucleus basalis of Meynert at autopsy, which explained the moderate increase in the [^18^F]PI-2620 PET signal in these regions. Across modalities, a strong association was observed between quantitative AT8 occupancy and autoradiography ratios (*R* = 0.878, *p* < 0.001), even when challenged by consideration of both target regions as cofactors across all samples (Fig. 4c). Furthermore, the quantitative AT8 occupancy (*R* = 0.584, *p* = 0.014) and autoradiography ratios (*R* = 0.556, *p* = 0.021) were consistent with the tau PET signals acquired 5–63 months before death in vivo (Fig. 4c).

### In vitro autoradiography confirms tau-positive neurons and oligodendrocytes as the major sources of tau tracer binding in tissues from patients with PSP

Next, we translated our murine cell type findings to human tau PET imaging and examined the detailed sources of tau PET signals. A correlation analysis between the area of AT8-positive neurons/oligodendrocytes (NFT/CB) and the area of AT8-positive astrocytes (TA, including TF) with [^18^F]PI-2620 autoradiography signals was performed using autopsy tissues derived from 16 patients with PSP (Supplemental Table 2). This PSP sample was selected based on absence of α-synuclein, TDP-43 or FUS copathology and limited β-amyloid copathology to minimize confounding factors, as assessed in the frontal cortex.

**Fig. 5:**
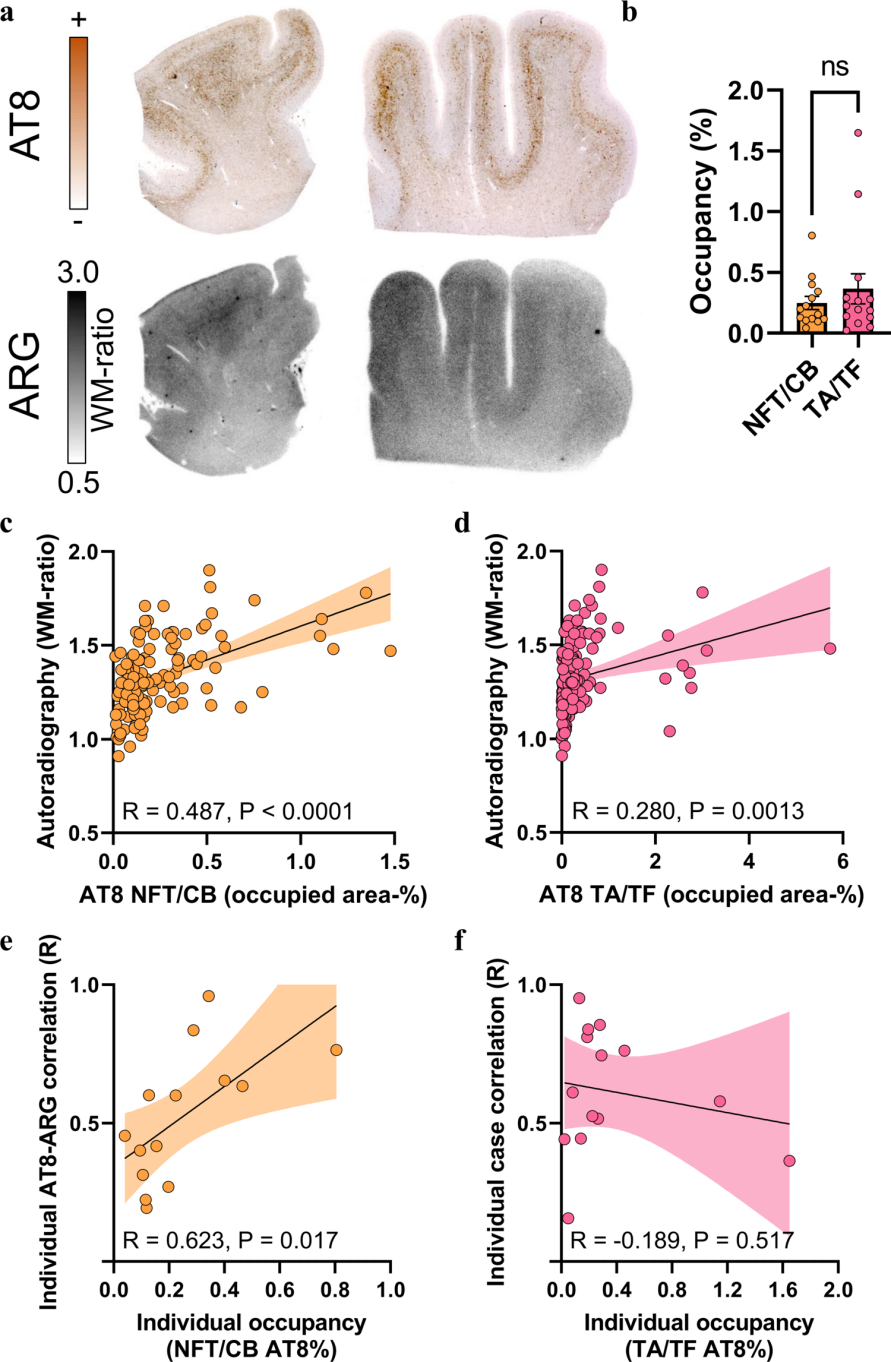
Cell type-specific immunohistochemistry-to-autoradiography correlation in the frontal medial gyrus of tissue samples from patients with PSP. **a** AT8 immunohistochemistry together with [^18^F]PI-2620 autoradiography of adjacent frontal medial gyrus sections from two exemplary patients with definite PSP. ARG = autoradiography. WM = white matter. **b** Comparison of neuronal/oligodendroglial (NFT/CB) and astrocytic (TA, including TF) AT8 occupancy in frontal medial gyrus sections from patients with definite PSP (*n* = 14). **c**, **d** Quantitative analysis of the correlation between tau abundance in the NFT/CB and TA/TF with autoradiography signals in the frontal medial gyrus subfields (including gray matter and white matter regions). Regression lines (including 95% confidence intervals) were calculated for 129 subfields derived from *n* = 14 patients with definite PSP after confirming the normality of the residuals. R indicates Pearson’s correlation coefficient. **e**, **f** Dependency of individual subfield correlation coefficients (AT8 × autoradiography) from the mean AT8 occupancy of all subfields per patient as determined for NFT/CB and TA/TF. Regression lines (including 95% confidence intervals) were calculated for 14 patients with definite PSP with normally distributed data. R indicates Pearson’s correlation coefficient

The frontal cortex was used as the primary brain region of interest due to the low probability of off-target sources [29]. Here, we found substantial visual agreement between AT8 immunohistochemistry and [^18^F]PI-2620 autoradiography signals in FFPE sections of patients with definite PSP (Fig. 5a). We tested the differential associations of neuronal/oligodendroglial (NFT/CB) and astrocytic (TA, including TF) tau abundances, as determined by AT8 immunohistochemistry (Fig. 5b), with [^18^F]PI-2620 autoradiography quantification in 129 predefined subfields of the gray matter and white matter (drawn on autoradiography sections by investigators blinded to the corresponding AT8 sections; Supplemental Fig. 4). NFT/CB tau abundance in PSP samples correlated more strongly with autoradiography signals (*R* = 0.487, *p* < 0.0001; Fig. 5c) than did TA/TF tau abundance (*R* = 0.280, *p* = 0.0013; Fig. 5d). A regression analysis with NFT/CB and TA/TF tau abundances as predictors revealed that only NFT/CB tau (*β* = 0.455, *p* < 0.0001) but not TA/TF tau (*β* = 0.068, *p* = 0.442) explained the [^18^F]PI-2620 autoradiography signal. We noticed that subfield-specific tau abundance in individual samples from patients with PSP was strongly correlated with autoradiography signals above only 0.2% of the mean occupied area of tau-positive NFT/CB (*R* = 0.623, *p* = 0.017; Fig. 5e), indicating the sensitivity threshold for translation into measurable signals. In contrast, individual samples from patients with PSP presenting high mean occupied areas of tau-positive TA/TF did not show strong individual subfield correlations (Fig. 5f), again suggesting the limited translation of TA/TF tau abundance to autoradiography and PET signals.

**Fig. 6:**
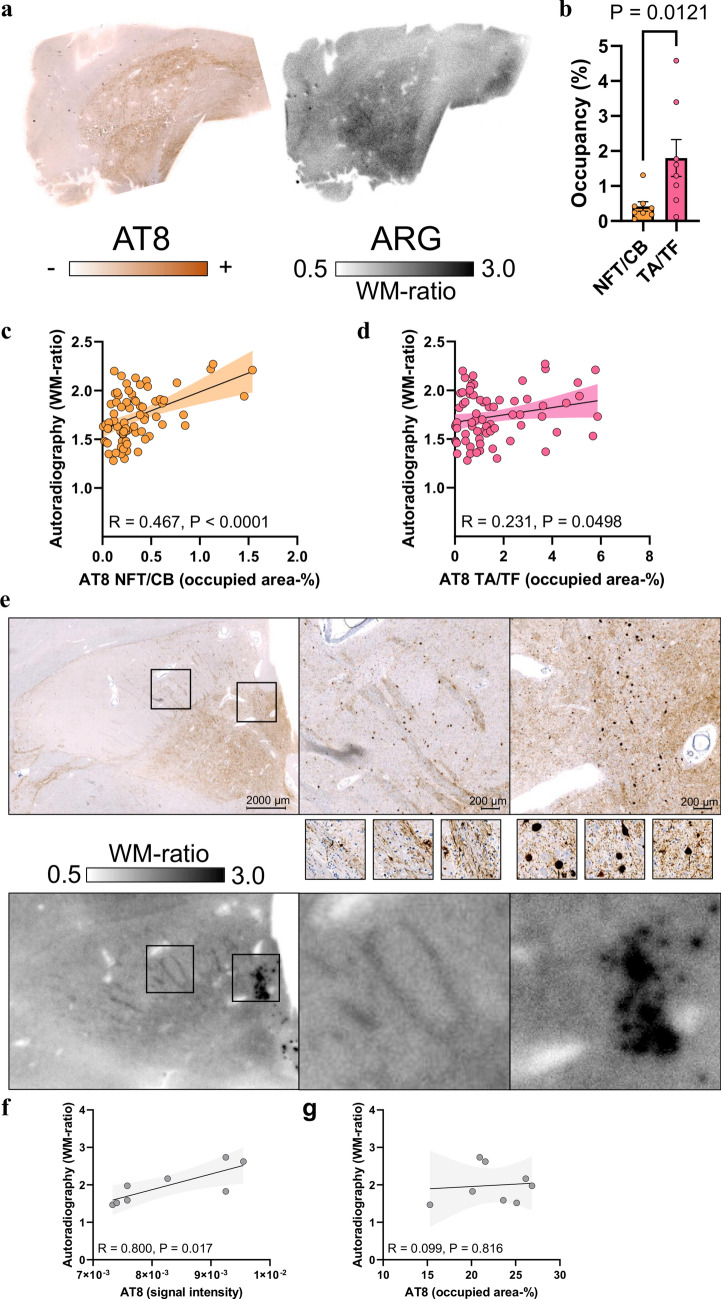
Cell- and substructure-specific immunohistochemistry-to-autoradiography correlations in the basal ganglia of tissue samples from patients with PSP. **a** AT8 immunohistochemistry together with [^18^F]PI-2620 autoradiography of the adjacent basal ganglia section from one exemplary patient with definite PSP. **b** Comparison of neuronal and oligodendroglial (NFT/CB) versus astrocytic (TA, including TF) AT8 occupancy in the globus pallidus of patients with definite PSP (*n* = 8). **c** Quantitative analysis of the correlation between NFT/CB tau abundance and autoradiography signals in the basal ganglia subfields. A regression line (including 95% confidence intervals) was calculated for 73 subfields derived from *n* = 8 patients with definite PSP. R indicates Pearson’s correlation coefficient. **d** Quantitative analysis of the correlation between TA/TF tau abundance and autoradiography signals in the basal ganglia subfields. A regression line (including 95% confidence intervals) was calculated for 73 subfields derived from *n* = 8 patients with definite PSP. R indicates Pearson’s correlation coefficient. **e**–**g** Quantitative analysis and visual correlation between AT8 intensity and AT8 occupancy with autoradiography signals in an individual sample from a patient with PSP. The small images in (E) show high-magnification images of faint AT8 intensity in white matter fibers and strong AT8 intensity in large neurons (NFTs). Note the strong autoradiography signal, which is discernible as single spots in the same area. Regression lines (including 95% confidence intervals) were calculated for *n* = 8 subfields of one patient with definite PSP. R indicates Pearson’s correlation coefficient

Despite substantial visual agreement in most samples from patients with PSP (Fig. 6a) and the presence of predominant TA/TF tau aggregation compared with sparse NFT/CB tau aggregation (Fig. 6b), only NFT/CB tau occupancy showed a substantial correlation with [^18^F]PI-2620 autoradiography signals in 73 subfields of the globus pallidus (Fig. 6c, *R* = 0.467, *p* < 0.0001). In contrast, the quantitative agreement between the TA/TF tau occupancy and [^18^F]PI-2620 autoradiography signals reached only borderline significance (Fig. 6d, *R* = 0.231, *p* = 0.0498). A regression model including NFT/CB AT8 occupancy and TA/TF AT8 occupancy as predictors provided a significant explanation of autoradiography signals only by NFT/CB tau (*β* = 0.676, *p* < 0.0001) but not by TA/TF tau (*β* = − 0.278, *p* = 0.081). Compared with the frontal cortex, AT8-positive areas in the basal ganglia were characterized by greater heterogeneity in AT8 staining intensity, including a high abundance of AT8-positive axons and occupancy of large neurons with high AT8 intensity in individual patients (see examples in Fig. 6e–g). Notably, large AT8-positive neurons (NFTs) were visually discernible in the [^18^F]PI-2620 autoradiography images (Fig. 6e). We investigated the impact of the AT8 density on the resulting autoradiography signal and observed a strong association between the AT8 lesion density and autoradiography signals in individual patients (*R* = 0.800, *p* = 0.017; Fig. 6f), whereas the AT8-occupied area was not a significant predictor of the autoradiography signal in these patients (Fig. 6g). Notably, some samples from patients with PSP presented a visually detectable [^18^F]PI-2620 autoradiography signal in the putamen, although AT8 occupancy was low, resulting in higher yet unexplained background [^18^F]PI-2620 signals in the putamen of healthy controls than in cortical areas [12]. We further performed tau isoform-specific staining using RD3 and RD4 antibodies and visually compared these results to AT8 staining. RD3 occupancy was barely detectable across all cases for both regions examined. In some frontal cortex sections, RD4 occupancy was also limited, leading to a nearly equal proportion of RD3 occupancy. Nevertheless, all the subjects exhibited a predominance of RD4-positive tau pathology compared with RD3-positive pathology in the frontal cortex, and RD4-positive tau pathology was even more pronounced in the basal ganglia (Supplemental Figs. 7 and 8). Importantly, significant tau accumulation was not detected in the frontal cortex or basal ganglia of the four deceased PD patients, as assessed by AT8 immunostaining and [^18^F]PI-2620 autoradiography. Additionally, only minor off-target binding was observed across PD patients (Supplemental Fig. 9).

### High oligodendroglial density at the boundary of gray and white matter indicates an improved cortical target region for tau PET in patients with PSP

**Fig. 7:**
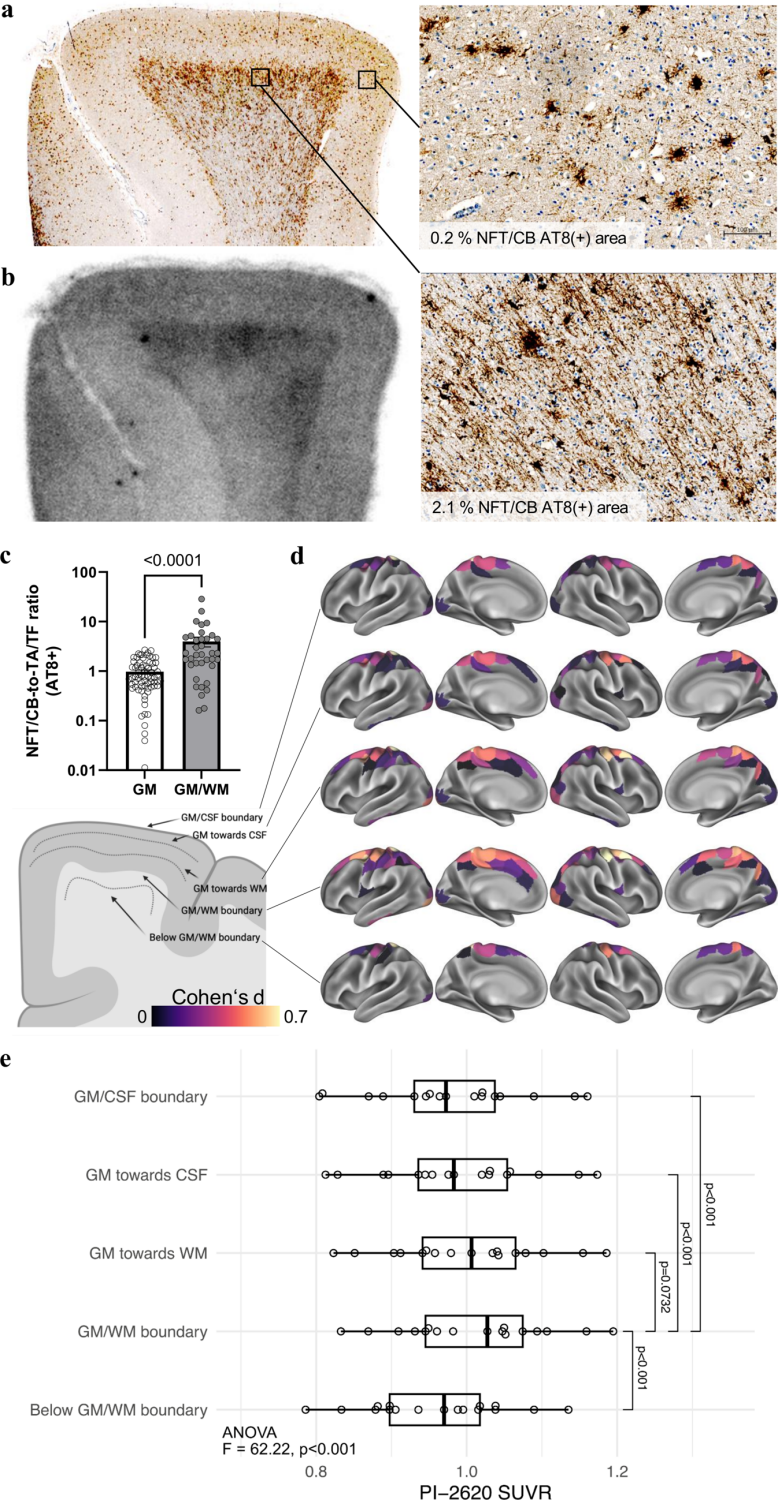
High oligodendroglial tau abundance at the GM/WM boundary facilitates the definition of an optimized frontal lobe target region in patients with PSP. **a**, **b** AT8 immunohistochemistry together with [^18^F]PI-2620 autoradiography of the adjacent frontal medial gyrus section from an exemplary patient with definite PSP. High-magnification images show high oligodendroglial tau levels in the boundary of gray matter and white matter, whereas cortical layers are characterized by low neuronal/oligodendroglial (NFT/CB) tau levels but abundant astrocytic (TA) tau inclusions. **c** Quantitative comparison of the NFT/CB-to-TA/TF ratios of tau occupancy in gray matter and the boundaries of gray matter and white matter. Data were derived from NFT/CB and TA/TF AT8 occupancy in the frontal medial gyrus subfields of patients with definite PSP (*n* = 14). **d** Schematic illustration of the PET SUVR assessment from the GM/WM boundary to the GM/CSF boundary. Surface renderings illustrate standardized group differences (Cohen’s d) between 17 PSP-RS patients and 9 healthy controls for the 200 regions of the Schaefer cortical atlas. Group differences are shown across 5 MRI-based cortical surface reconstructions applied to the PET data, systematically shifted from the GM/CSF boundary toward the GM/WM boundary and below. **e** Boxplots showing the corresponding PET SUVRs in the 17 PSP-RS patients; using repeated-measures ANOVA, followed by post hoc pairwise comparisons with Tukey's test for multiple comparisons

Finally, we exploited the cellular and regional findings of our study to further improve the use of tau PET for the diagnosis of 4R-tauopathies. Pronounced regional autoradiography signals were observed in white matter areas adjacent to the GM/WM boundary (Fig. 7a). Consistent with previous reports [15, 16], these areas were characterized by distinctly higher NFT/CB-to-TA/TF ratios than cortical gray matter regions (fourfold, *p* < 0.0001; Fig. 7c). We assessed the relevance of this observation in vivo by analyzing seventeen [^18^F]PI-2620 tau PET scans of patients with PSP-RS and a high likelihood of underlying 4R-tauopathy and nine healthy controls with an MRI-based layer segmentation of gray matter and WM/GM boundary areas of the frontal cortex (Supplemental Fig. 10). Here, we found a greater effect size for the comparison of tau PET signals in the frontal cortex of patients with 4R-tauopathies and controls using the GM/WM boundary target region (Cohen’s *d* = 1.68) in contrast to the common gray matter target region (Cohen’s *d* = 1.37). Thus, focusing on oligodendroglia-rich regions with high AT8 positivity enhanced the assessment and diagnostic accuracy of cortical tau burden by [^18^F]PI-2620 tau PET in patients with 4R-tauopathies.

## Discussion

In this translational study, we used a large spectrum of methodological approaches, including innovative scRadiotracing and a cell-type-specific correlation of autoradiography signals, to disentangle the discrepant findings of previous reports that investigated second-generation tau PET in 4R-tauopathies. As a major achievement, we detected elevated radiotracer binding in isolated neurons after in vivo injection in mice. Furthermore, our data indicate that tau PET signals in individuals with 4R-tauopathies are driven by dense neuronal and oligodendroglial tau aggregation, whereas faint tau-positive structures of astrocytes and tau fragments are not capable of translating radiotracer binding into in vivo signals. Finally, we provide the first [^18^F]PI-2620 PET-to-autopsy correlation and show that cortical tau PET signals deserve optimized target regions at the boundary between gray and white matter.

The overarching research question of this work addressed the validity of second-generation tau PET signals in individuals with 4R-tauopathies. A recent blocking study revealed that [^3^H]PI-2620, but not [^3^H]MK-6240 or [^3^H]RO-948, exhibited high specific binding in the frontal cortex of deceased patients with PSP and CBD [32]. In contrast, another recent autoradiography head-to-head comparison did not detect a significant extent of [^18^F]AV-1451, [^18^F]MK-6240, or [^18^F]PI-2620 binding to non-AD tauopathies [1]. Such discrepancies were previously reported for [^18^F]AV-1451, which presented positive [31] and negative [33] autoradiography signals in brain sections from individuals with 4R-tauopathy. However, in addition, in vitro saturation assays and competitive binding assays [52], as well as molecular docking [28], have provided additional evidence that PI-2620 binds to 4R-tau. Therefore, we performed a battery of experiments to test the translation of PI-2620 binding to in vivo PET signals.

First, we showed that second-generation tau PET with [^18^F]PI-2620, a radiotracer that has affinity for 3- and 4-repeat tau, has sufficient sensitivity to detect the accumulation of tau pathology in transgenic PS19 mice. Tau PET indicated earlier sensitivity for pathological alterations than 3 T structural MRI-based atrophy measures did, but we note that both modalities could still be improved in terms of resolution, i.e., the availability of ultrahigh-field MRI. We acknowledge that PET signal spill-over from adjacent intra- and extracerebral structures may have influenced our findings [34, 35]. Both WT and PS19 mice were analyzed using the same standardized approach to mitigate this spill-over [38]. Additionally, we selected a tau-negative reference region in the central striatum and target regions in the entorhinal cortex and brainstem, positioned at a sufficient distance from the edges of the PET images. In terms of preclinical tau PET imaging, [^18^F]PM-PBB3 also showed elevated tau PET signals in the rTg4510 mouse model, which suggests that in vivo monitoring of 4-repeat tau pathology in tau models is feasible with next-generation tau radiotracers [49]. From a biomechanical perspective, high individual tau PET levels were associated with greater atrophy in our cohort of PS19 mice, which highlights the link between tau and neurodegeneration. Furthermore, this finding shows that [^18^F]PI-2620 tau PET signals in PS19 mice may still be underestimated, since an individual MRI-based partial volume effect correction has yet to be established in mice. Nevertheless, while the PS19 model is based on a mutation linked to FTD, it remains relevant for studying certain aspects of 4R-tauopathies, as both patients with PSP and PS19 mice exhibit hyperphosphorylated 4R-tau leading to neurodegeneration [3]. However, the PS19 model primarily reflects neuronal tau pathology, lacking the strong glial involvement observed in patients with PSP. Alternative models, such as seed-based approaches [4], the use of tau fibrils from PSP patients [14, 43], or AAV-mediated tau aggregation [50], can be used to address this discrepancy, because they provide a more accurate representation by inducing tau pathology in both neurons and glial cells, thereby better replicating the complexity of PSP [36].

One major goal of our investigation was to determine whether tau PET signals are derived from tau-positive cells. In this context, earlier tau PET radiotracers such as [^18^F]THK5117 and [^18^F]AV1451 also presented increased signals in P301S or BiGT mice [13], but due to the identified off-target binding to monoamine oxidases [37], the observed increased signals could also be derived from activated immune cells, i.e., reactive astrocytes. As a major novel approach, we applied cell sorting after in vivo tracer injection [7, 8, 56] to a tau radiotracer. Here, we found that (i) tau tracer binding was much higher in neurons than in astrocytes, (ii) neurons from PS19 mice had nearly twofold higher tracer uptake than neurons from wild-type mice, and (iii) single-cell tracer uptake was used to determine the in vivo tau PET signal. Thus, scRadiotracing directly links cellular binding to translation toward an in vivo tau PET signal. Notably, our data cannot prove the occurrence of intraneuronal binding to 4R-tau, but we deem neuronal off-target sources less likely than immune cells [37], vessels, iron-associated regions, calcifications in the choroid plexus, or leptomeningeal melanin [31]. Interestingly, we also observed greater tracer uptake by neurons than astrocytes in wild-type mice, which could indicate larger cell body volumes of neurons that subsequently contain higher levels of tau under physiological conditions than astrocytes do [54]. scRadiotracing data from PS19 mice were consistent with the results of immunohistochemistry, which also revealed neurons as the major tau-positive cell type. Due to the decay of radioactivity over time and the lengthy procedure (6–7 h), we established that valid results required signal-to-noise ratios above 2.0. Using the tau tracer [^18^F]PI-2620 and the protocol outlined, we estimate the lower detection limit for the tracer to be approximately 1–2 × 10^4^ astrocytes, whereas the samples analyzed in this study were consistently > 10^5^. Additionally, astrocyte tracer uptake was measured prior to neuronal uptake, thereby ruling out any sensitivity issues for astrocyte detection. As a result, despite higher tracer uptake in neurons, the radioactivity measurement remained sufficient, even for lower numbers of isolated neurons compared to astrocytes. As a limitation, our methodology did not have sufficient sensitivity to analyze tau tracer uptake in cells other than neurons and astrocytes (i.e., vascular cells and oligodendrocytes). Future work could optimize the sensitivity of scRadiotracing and directly correlate cellular radiotracer binding with the cellular amount of the tau protein [7].

Our study included the first sample of deceased patients with 4R-tauopathies and disease controls, which allowed us to establish a PET-to-autopsy correlation between in vivo [^18^F]PI-2620 signals and the quantitative tau load ex vivo. The variable time intervals between PET imaging and death need to be considered a limitation, since the tau load could still change after PET imaging. Longitudinal tau PET scans in PSP patients could overcome this challenge and provide critical insights into the progression of in vivo tau pathology and may help predict the disease trajectory in individual patients. Nevertheless, we obtained a strong correlation between in vivo PET signals and AT8 occupancy in the frontal cortex and the basal ganglia in our cohort consisting of seven patients with PSP and two disease controls. In vivo tau PET signals in patients without evidence of significant tau pathology ex vivo matched the signal levels reported for healthy individuals and disease controls [12]. Notably, one patient with definite PSP at autopsy was diagnosed with nonfluent primary progressive aphasia 3.2 years before death, and one patient with definite PSP at autopsy was diagnosed with behavioral variant frontotemporal dementia 2.5 years before death. However, both also showed a PSP-like [^18^F]PI-2620 PET signal in the basal ganglia at the time of their diagnostic workup, which highlights the diagnostic value of PET imaging when 4R-tauopathies are a possible differential diagnosis. Furthermore, [^18^F]PI-2620 PET also reliably captured AT8-positive tau (co)pathology of the globus pallidus in the included TBK1 carrier. Synucleinopathies, such as PD and Lewy body dementia (DLB) [5], are among the most common differential diagnoses for PSP [23]. Consistent with the recent literature reporting an absence of postmortem tau tracer binding in Lewy body-containing tissues, our postmortem analysis similarly revealed no significant AT8 occupancy or [^18^F]PI-2620 autoradiography signal in the frontal cortex and basal ganglia of the four PD patients examined [1, 2]. In addition to previously identified sources of off-target binding, these findings highlight the potential of [^18^F]PI-2620 in differentiating 4R-tauopathies from α-synucleinopathies. Specifically, the results confirmed the absence of relevant off-target binding to α-synuclein-related pathology. Nevertheless, we note that a limited degree of off-target/unspecific binding in the basal ganglia, as indicated by [^18^F]PI-2620 autoradiography, should still be considered for image interpretation. In particular, regions exhibiting elevated in vivo binding of [^18^F]PI-2620 in PSP correspond to off-target areas previously identified with other tau ligands in healthy and disease controls [29].

We determined the contributions of neuronal, oligodendroglial and astrocytic tau to human autoradiography signals using a dataset of patients with PSP presenting with very limited copathology to overcome the limitations of mixed pathology and variable intervals between PET and autopsy. Previously, we reported greater agreement between postmortem neuronal tau covariance and tau PET covariance than between astrocytic and oligodendroglial tau covariance in the same samples from patients with PSP [19]. Building upon these results, we observed greater agreement between the neuronal/oligodendroglial tau abundance and autoradiography signals than the astrocytic tau abundance in the frontal cortex. The most likely explanation for this finding was provided by a similar analysis of the basal ganglia. Here, regions with neighboring large dense neurons even resulted in focal autoradiography signals, whereas astrocyte- or axon-dominated regions with a lower density of AT8 revealed only lower increases in the signal.

Similar effects were observed for oligodendroglia with a higher AT8 density at the GM/WM boundary than for cortical astrocytes with a lower AT8 density. Thus, the faint processes of astrocytes and axon bundles likely suffer from partial volume effects within the tissue compartment, which hampers translation into a measurable tau radiotracer signal in vivo.

This finding aligns with our tau isoform-specific staining using RD3 and RD4 antibodies in the frontal cortex and basal ganglia. Consistently higher proportions of RD4 staining were observed in the frontal cortex and were even more pronounced in the basal ganglia of PSP patients, where RD4 occupancy accounted for the vast majority of the tau load. In particular, in cortical sections, we noticed pronounced autoradiography signals at the GM/WM boundary, and we were able to correlate these findings with high proportions of AT8-positive oligodendroglia in this particular region. A high oligodendroglial tau load in the white matter at the GM/WM boundary in patients with 4R-tauopathies has also been reported in larger autopsy studies [16]. In vivo transfer of this observation to [^18^F]PI-2620 tau PET signals in patients with PSP revealed the clinical relevance of this regional predominance, with higher effect sizes of PET signals extracted from the GM/WM boundary than from the whole cortex region (including gray matter and white matter). This result should guide optimized frontal cortex target region selection for tau PET analysis in patients with 4R-tauopathies to increase PET sensitivity for cortical tau. In addition, compared with static imaging, dynamic imaging has a greater probability of detecting clinically diagnosed 4R-tauopathies in vivo [12] [11]. This finding is also supported by a lack of in vivo signals in patients with PSP at later static imaging windows [51] and a decrease in target signals with imaging time [45], supporting the preference for early [46] or dynamic scanning [12] over late imaging windows. Thus, optimized target and reference tissues should be considered together with dynamic imaging to exploit the full diagnostic value of [^18^F]PI-2620 tau PET in patients with 4R-tauopathies.

In summary, we show that aggregated neuronal and oligodendroglial 4R-tau translates to measurable tau PET signals in patients with PSP and CBS, whereas astrocytic and axonal tau inclusions are a minor source of in vivo PET signals. Our novel approach of cell sorting after radiotracer injection can be readily used to test the cell type specificity of novel radiotracers with 4R-tau affinity.

## Supplementary Information

Below is the link to the electronic supplementary material.

Supplementary file1 (DOCX 11283 KB)

## Data Availability

All the data needed to evaluate the conclusions in Figs. 1–7 are presented in the paper and/or the Supplementary Materials. Imaging data will be shared in DICOM format upon reasonable request to the corresponding author.
