# Supplementary material for: Neuronal and oligodendroglial, but not astroglial, tau translates to in vivo tau PET signals in individuals with primary tauopathies
Source: Acta Neuropathol. 2024 Nov 24;148(1):70. doi: 10.1007/s00401-024-02834-7 (PMC11586312; doi:10.1007/s00401-024-02834-7)
Supplement: Supplementary file 1 — Supplementary file1 (DOCX 11283 KB) [file 401_2024_2834_MOESM1_ESM.docx]

**Supplement to Neuronal and oligodendroglial, but not astroglial, tau translates to in vivo tau PET signals in individuals with primary tauopathies**

Luna Slemann, Johannes Gnörich, Selina Hummel, Laura M. Bartos, Carolin Klaus, Agnes Kling, Julia Kusche-Palenga, Sebastian T. Kunte, Lea H. Kunze, Amelie Englert, Sabrina Katzdobler, Carla Palleis, Alexander Bernhardt, Alexander Jäck, Andreas Zwergal, Franziska Hopfner, Sebastian Römer, Gloria Biechele, Sophia Stöcklein, Gerard Bischof, Thilo van Eimeren, Alexander Drzezga, Osama Sabri, Henryk Barthel, Gesine Respondek, Timo Grimmer, Johannes Levin, Jochen Herms, Lars Paeger, Marie Willroider, Leonie Beyer, Günter U. Höglinger, Sigrun Roeber, Nicolai Franzmeier, and Matthias Brendel

**Content**

**Supplemental Figure 1–Methodological details of small animal PET/MRI**

**Supplemental Figure 2–Methodological details of AT8 immunohistochemical segmentation**

**Supplemental Figure 3–Sample size estimation for the assessments of the tau burden and atrophy by small animal PET/MRI**

**Supplemental Figure 4–Subfield definitions in autoradiography and AT8-stained sections**

**Supplemental Figure 5–Overview of [^18^F]PI-2620 PET scans in individual PS19 and WT mice**

**Supplemental Figure 6–Correlations between the tau burden and tau PET signals in PS19 mice**

**Supplemental Figure 7–Comparison of AT8, RD3 and RD4 immunoreactivity in the frontal cortex**

**Supplemental Figure 8–Comparison of AT8, RD3 and RD4 immunoreactivity in the basal ganglia**

**Supplemental Figure 9–Immunohistochemistry and autoradiography of autopsy samples from patients with PD**

**Supplemental Figure 10–Representative [^18^F]PI-2620 PET images for illustration of GM/WM segmentation**

**Supplemental Table 1–Overview of PET samples from the autopsy cohort**

**Supplemental Table 2–Overview of samples from the autoradiography cohort of patients with PSP**

**Supplemental Table 3–Overview of samples from the autopsy cohort of patients with PD**

**Supplemental Figure 1**


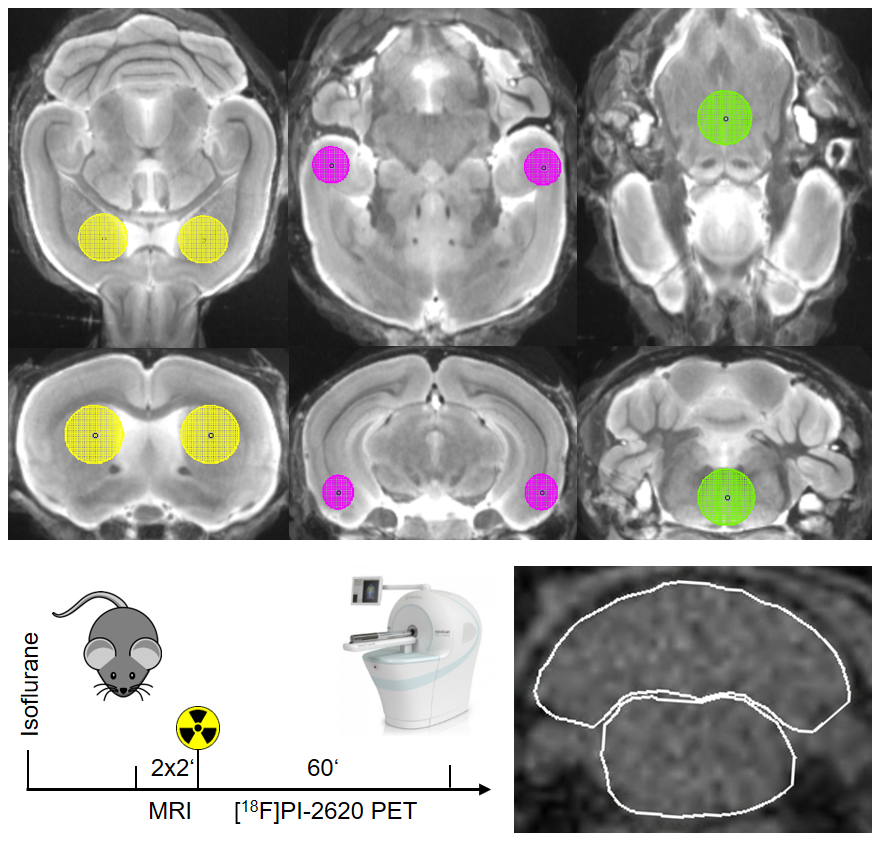


**Supplemental Figure 1–Methodological details of small animal PET/MRI.** The upper panel shows the definitions of the PET target (brainstem = green, entorhinal cortex = purple) and reference (striatum = yellow) regions. The lower panel shows the timing of the PET/MRI acquisition protocol and the delineation of brainstem and cerebellum volumes in the coronal plane.

**Supplemental Figure 2**

**
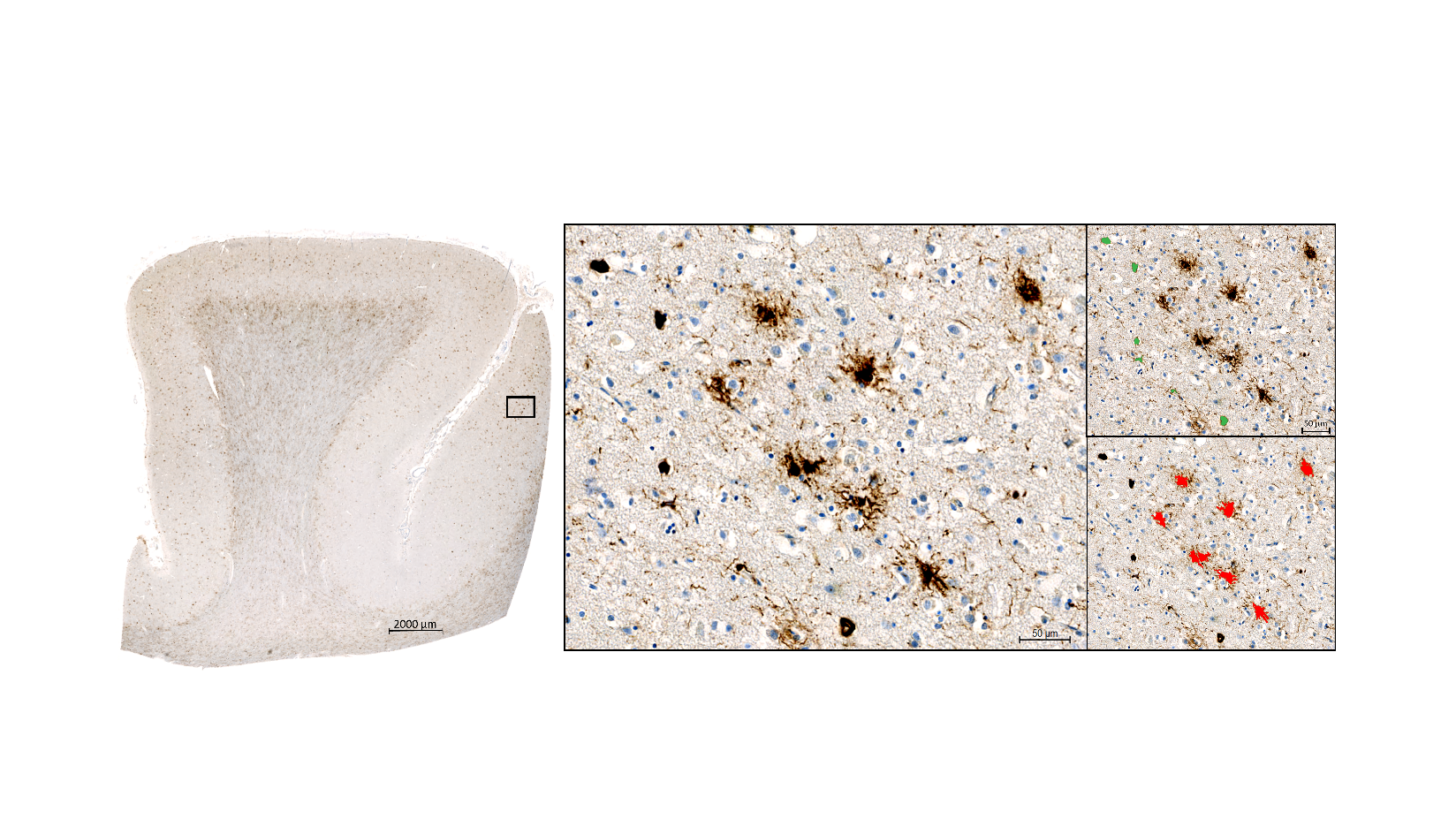
**

**Supplemental Figure 2–Methodological details of AT8 immunohistochemical segmentation.** Overview of an exemplary AT8-stained frontal cortex section, followed by detailed high-magnification views. The upper high-magnification image shows AT8-positive NFTs and CBs (green), whereas the lower high-magnification image highlights AT8-positive TAs (red). Consistent masking and automated detection were applied across all samples using ZEN 3.4 blue edition software (Zeiss, Jena, Germany) to ensure accurate and reproducible quantification.

**Supplemental Figure 3**

**
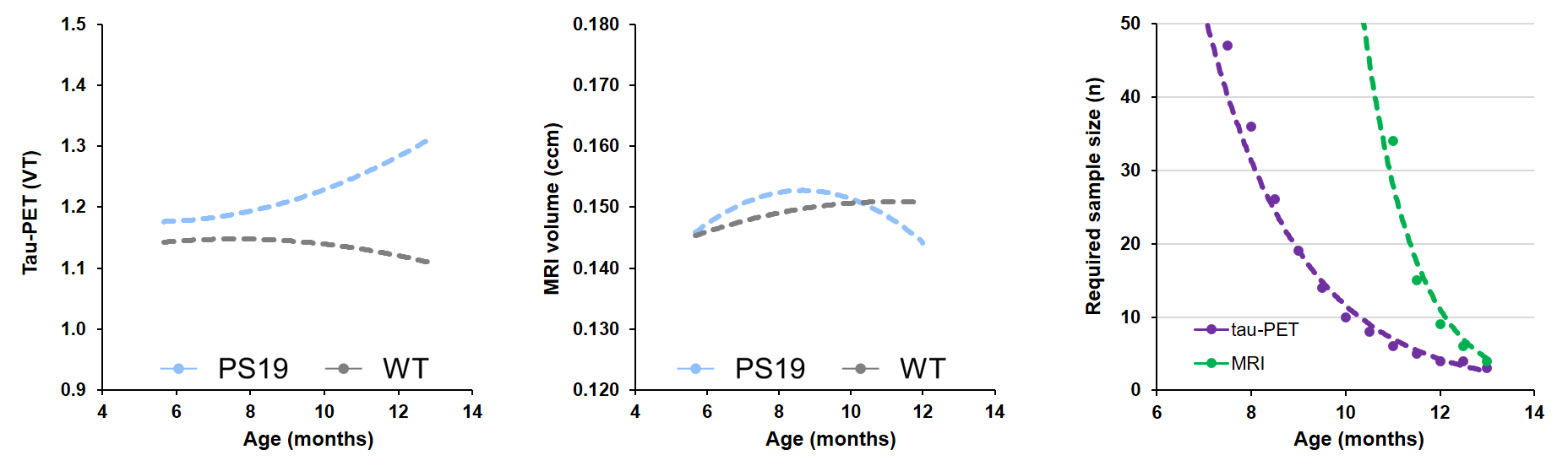
**

**Supplemental Figure 3–Sample size estimation for the assessments of the tau burden and atrophy by small animal PET/MRI.** Trajectories of tau PET signals and brain volumes as a function of age in PS19 and wild-type (WT) mice together with the required sample size to detect significant differences with each modality at a power of 0.8 and an alpha of 0.05.

**Supplemental Figure 4**


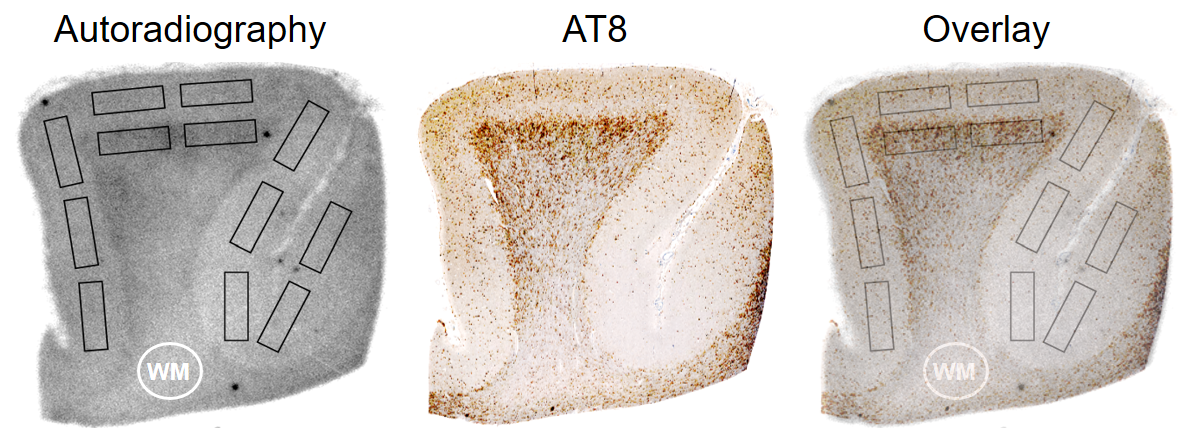


**Supplemental Figure 4–Subfield definitions in autoradiography and AT8-stained sections.** Representative images showing subfield definitions in the gray matter and subcortical white matter of the medial frontal gyrus from a patient with definite PSP detected using autoradiography (left panel), the corresponding AT8-stained section (middle panel) and the overlay (right panel). The circular white matter (WM) region shows the reference region for the determination of autoradiography target-to-white matter ratios. In this example, both subfields in the autoradiography center were defined as gray matter/white matter boundaries, which showed a high abundance of coiled bodies.

**Supplemental Figure 5**

**
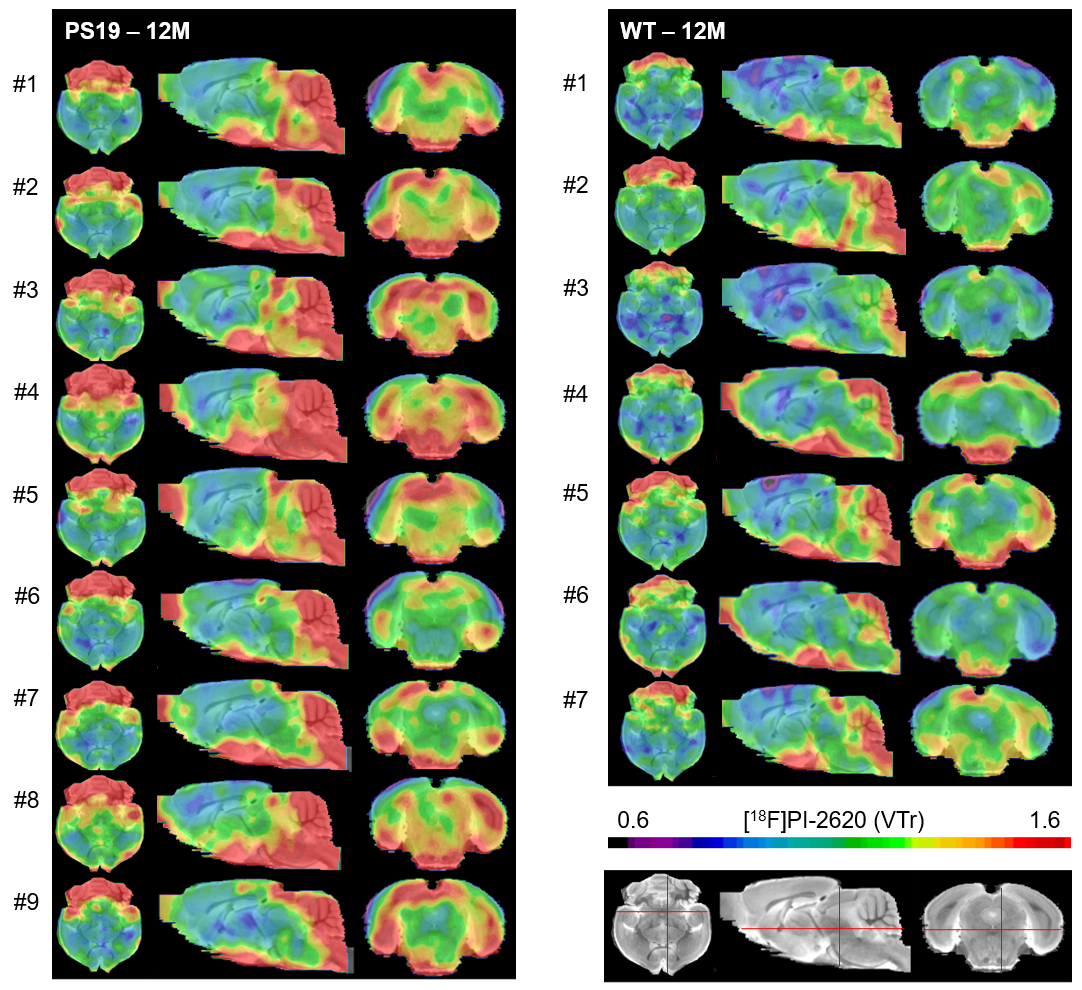
**

**Supplemental Figure 5–Overview of [^18^F]PI-2620 PET scans of individual PS19 and WT mice.** The comparison of individual axial, sagittal and coronal [^18^F]PI-2620 PET images (volume of distribution ratios, VT; striatal reference) overlaid on an MRI template shows radiotracer binding in transgenic (TG, n=9) and wild-type (WT, n=7) mice at 12 months of age.

**Supplemental Figure 6**

**
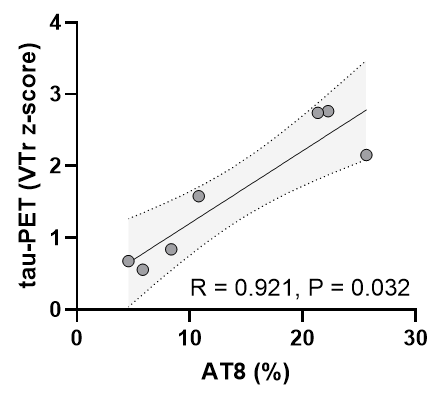
**

**Supplemental Figure 6–Correlations between the tau burden and tau PET signals in PS19 mice.** The plot shows matched region pairs of changes in AT8 area % and [^18^F]PI-2620 tau PET (volume of distribution ratio z scores) in the frontal cortex (n=4) and hippocampus (n=3) of PS19 mice.

**Supplemental Figure 7**

**
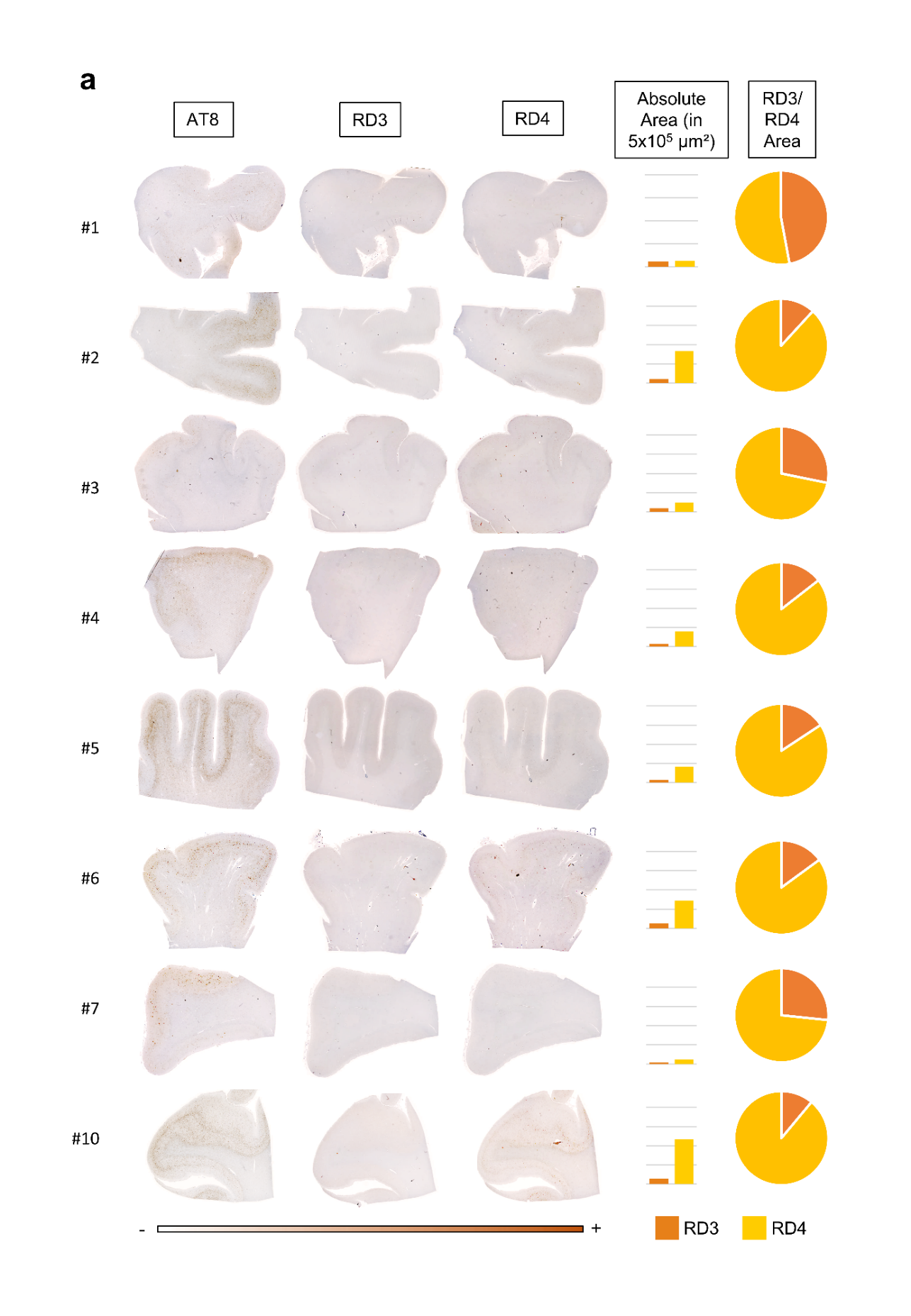
**

**
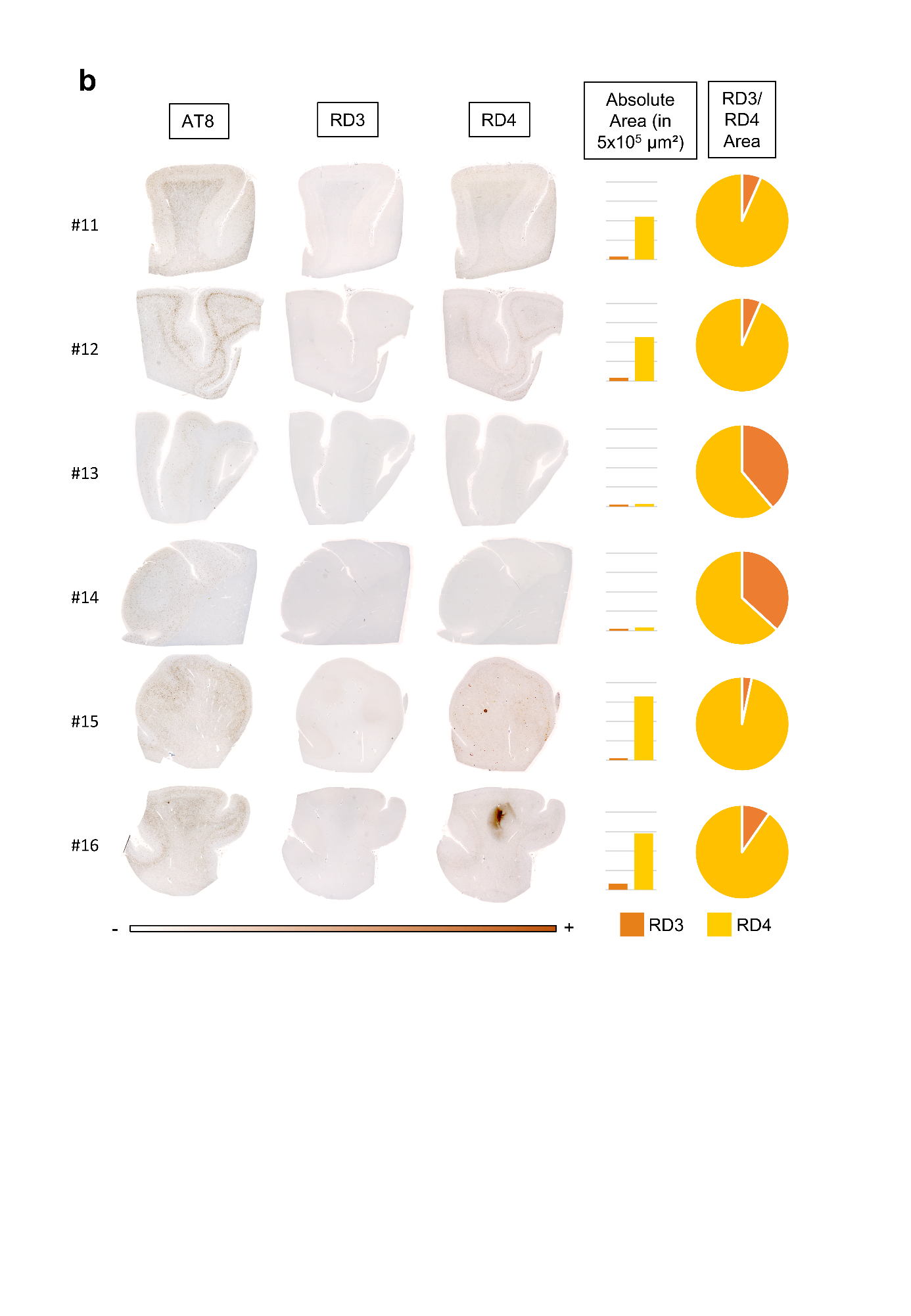
**

**Supplemental Figure 7–Comparison of AT8, RD3 and RD4 immunoreactivity in the frontal cortex.** (**A-B**) AT8, RD3 and RD4 immunohistochemical staining of adjacent sections of the frontal medial gyrus. The bar graphs depict the coverage for each tau antibody. Pie charts illustrate the compositions of RD3 and RD4 positivity.

**Supplemental Figure 8**

**
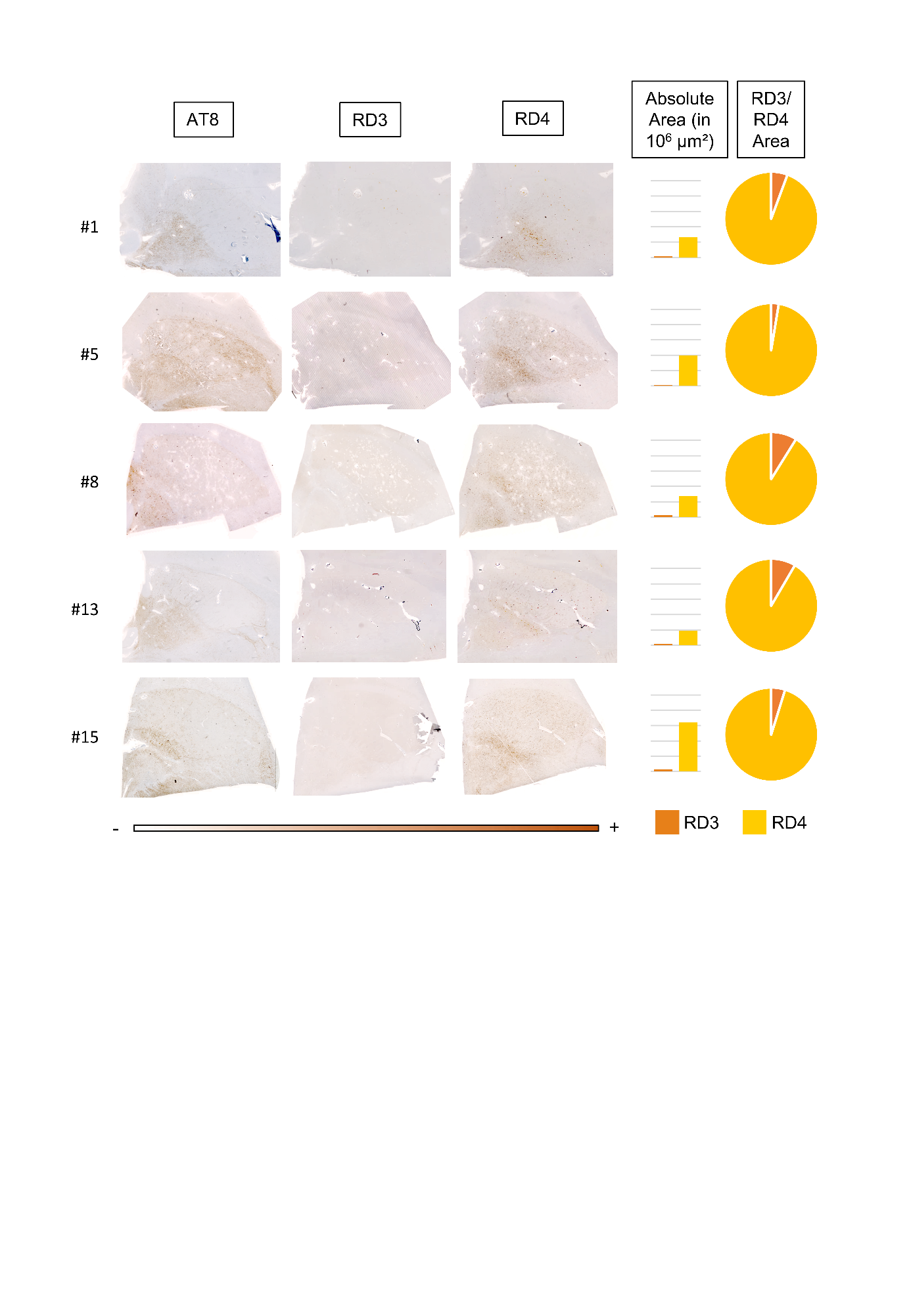
**

**Supplemental Figure 8–Comparison of AT8, RD3 and RD4 immunoreactivity in the basal ganglia.** (**A-B**) AT8, RD3 and RD4 immunohistochemical staining of adjacent sections of the basal ganglia. The bar graphs depict the coverage for each tau antibody. Pie charts illustrate the compositions of RD3 and RD4 positivity.

**Supplemental Figure 9**


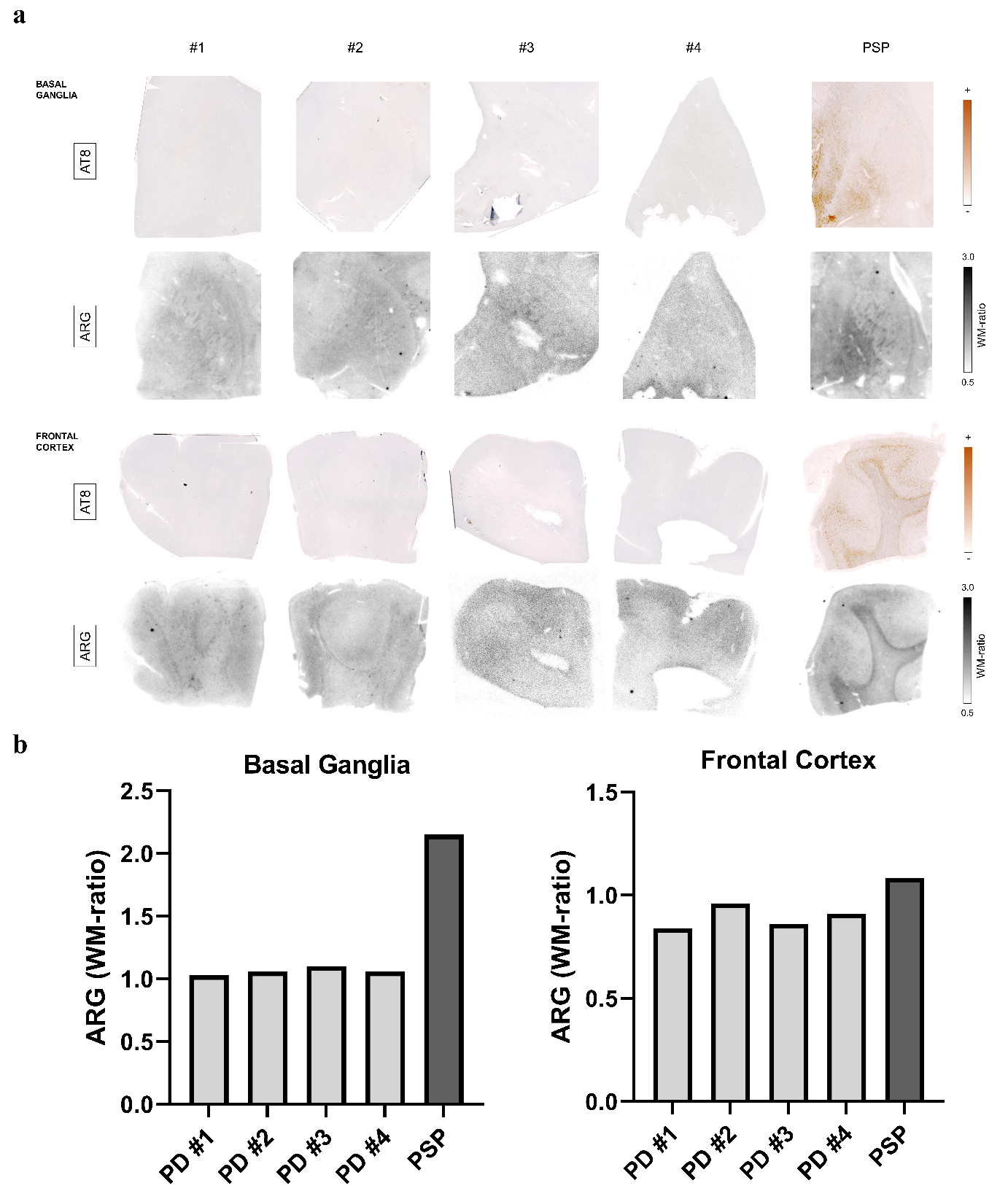


**Supplemental Figure 9–Immunohistochemistry and autoradiography of autopsy samples from PD patients.** (**A**) Basal ganglia and frontal cortex AT8 immunohistochemistry together with [^18^F]PI-2620 autoradiography in four patients with clinically diagnosed Parkinson’s disease (PD). ARG = autoradiography. Tissues from PSP patient #6 (see **Fig. 4**) are displayed on the right for comparison. (**B**) Bar graphs depict semi-quantitative autoradiography signal of the basal ganglia and the frontal cortex.

**Supplemental Figure 10**

**
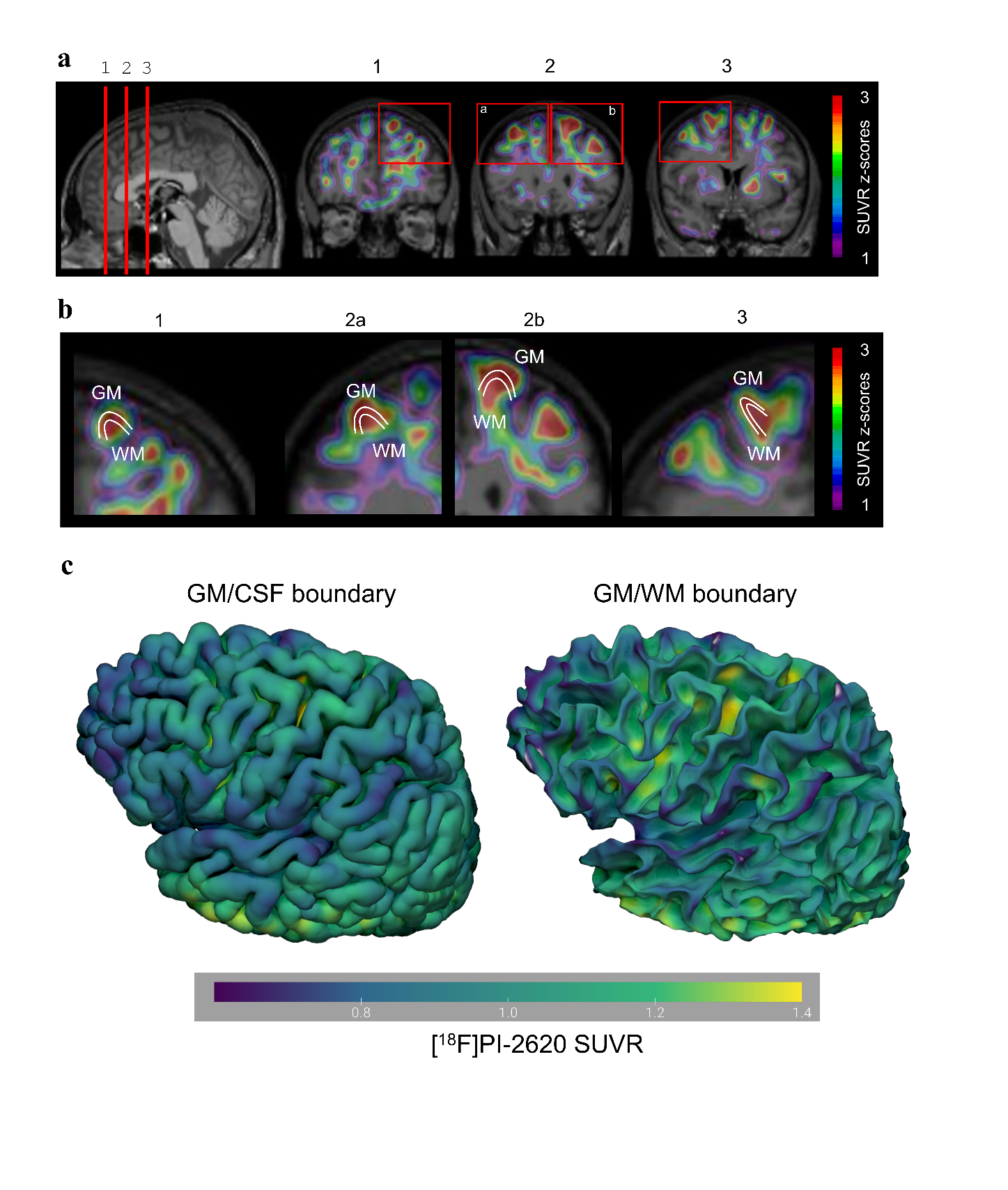
**

**Supplemental Figure 10–Representative [^18^F]PI-2620 PET images for illustration of GM/WM segmentation.** (**A**) Exemplary coronal slices of [^18^F]PI-2620 PET SUVR z-score images overlaid on a T1-weighted MRI. Red lines depict coronal cross sections. Red boxes represent zoomed-in images illustrated in (**B**). White lines depict the GM/WM boundaries in the frontal cortex of both hemispheres. (**C**) Representative 3D illustrations of [^18^F]PI-2620 PET SUVR uptake at the GM/CSF and GM/WM boundaries.

**Supplemental Table 1**

| **Case** | **Demographics** | | | | | **Diagnosis** | | | **Autopsy determinants** | | | **Copathology** | |
| --- | --- | --- | --- | --- | --- | --- | --- | --- | --- | --- | --- | --- | --- |
|  | Sex | Age at PET (y) | Age at  death (y) | Disease  duration (y) | Cause of death (death certificate) | Clinical  diagnosis | Clinical presentation | Autopsy  diagnosis | Brain  weight (g) | Postmortem  delay (h) | Fixation  time (d) | Aβ/α-syn/TDP-43/FUS | Frontal  cortex |
| #1 | female | 68 | 70 | 6 | Dysphagia | PSP-RS | Vertical gaze palsy, postural instability, axially emphasized hypokinetic-rigid syndrome, frontal brain signs | PSP, CAA, AD (B&B 3), AGD | 1182 | 42 | 76 |  | β-Amyloid |
| #2 | male | 64 | 66 | 4 | n.a. | PSP-RS | Axially emphasized hypokinetic-rigid syndrome with postural instability, vertical gaze palsy and generalized saccadic slowing, cognitive deficits and frontal brain syndrome | PSP, ARTAG, intranuclear inclusions of unclear etiology, discrete TDP-43 in the brainstem | 1363 | 90 | 31 |  | - |
| #3 | male | 70 | 71 | 6 | Atypical PD/recurrent pneumonia | PSP | Vertical gaze palsy, slowed saccades, macro square wave jerks, falls, akinetic-rigid Parkinson's syndrome, dysexecutive syndrome | PSP | 1570 | 39 | 63 |  | β-Amyloid |
| #4 | female | 73 | 77 | 6 | Cardiac arrhythmia | PSP-RS | Vertical gaze palsy, postural instability with a tendency to fall and retropulsion tendency, dysphagia, dysarthria, bilateral limb apraxia, buccofacial apraxia, intermittent diplopia | PSP, AGD, ARTAG, subdural hemorrhage, AD (B&B 1) | 1170 | 25 | 71 |  | β-Amyloid |
| #5 | female | 67 | 72 | 8 | Exsiccosis due to food refusal | nfvPPA DD: PSP-SL | Dysarthria, impaired verbal and visual memory, deficits in word fluency and visuoconstructive abilities, vertical gaze palsy**,** postural instability | PSP, AGD, AD (B&B 2) | 970 | 40 | 32 |  | β -Amyloid (very little) |
| #6 | male | 72 | 75 | 7 | Heart failure, COVID-19 | bvFTD/nfvPPA | n.a. | PSP | 1262 | 34-58 | 124 |  | n.a. |
| #7 | male | 63 | 67 | 4 | n.a. | nfPPA | n.a. | PSP, AD (B&B 3), mild AGD | 1512 | 30 | 48 |  | - |
| #8 | female | 65 | 65 | 1 | ALS-FTD | ALS-FTD, PPA | Bulbar deficits (dysphagia, fibrillation and atrophy of the tongue), atrophy of the thenar and hypothenar muscles on both sides (in the sense of damage to the 2nd motor neuron), increased masseter reflex (in the sense of damage to the 1st motor neuron) | FTLD/MND-TDP, AD (B&B 3), AGD, | 1261 | 48 | 88 |  | - |
| #9 | female | 75 | 75 | 6 | Loss of food and fluids | bvFTD (TBK1-mutation) | Dementia syndrome with changes in behavior (disinhibitory behavioral patterns, loss of empathy, sluggishness, stereotypies) and short-term memory impairment | FTLD-TDP (Type A),  LBD (Braak stage 5, neocortical),  AGD, ARTAG, AD/PART (B&B 2), central pontine myelinolysis,  hippocampal sclerosis,  multiple microinfarcts | 1080 | 18 | 40 |  | Synuclein, tau |

**Supplemental Table 1–Overview of PET samples from the autopsy cohort.** y = years, PD = Parkinson’s disease, ALS = amyotrophic lateral sclerosis, FTD = frontotemporal dementia, PSP-RS = progressive supranuclear palsy Richardson syndrome, nf = nonfluent, PPA = primary progressive aphasia, bv = behavioral variant, AD = Alzheimer’s disease, CAA = cerebral amyloid angiopathy, AGD = agyrophilic grain disease, ARTAG = aging-related tau astrogliopathy, B&B = Braak and Braak, TDP-43 = TAR DNA-binding protein 43, MND = motor neuron disease, PART = primary age-related tauopathy, Aβ = β-amyloid, APOE = apolipoprotein E, α-syn = alpha synuclein, FUS = fused in sarcoma, n.a. = not available, "-" = negative, copathology: FUS not examined

**Supplemental Table 2**

| **Case** | **Demographics** | | | | **Diagnosis** | | **Autopsy determinants** | | | **Copathology** | | |
| --- | --- | --- | --- | --- | --- | --- | --- | --- | --- | --- | --- | --- |
|  | Sex | Age at  death (y) | Disease  duration (y) | Cause of death | Clinical  diagnosis | Autopsy  diagnosis | Brain  weight (g) | Postmortem  delay (h) | Fixation  time (d) | Aβ/α-syn/TDP-43/FUS | Frontal  cortex | APOE |
| #1 | female | 67.3 | 7 | n.a. | PSP-RS | PSP, LBD (Braak stage 4), CAA | 1290 | 14.27 | 646 |  | Aβ: vascular deposits | n.a. |
| #2 | female | 70.9 | 5 | likely pulmonary embolism | CBS | PSP, AD (B&B stage2, Thal 2, CERAD 0), mild arteriosclerosis | 1086 | 49.67 | 325 |  | Aβ: some plaques | n.a. |
| #3 | female | 74.9 | 10 | aspiration pneumonia | CBS | PSP, CAA | 1180 | 25.50 | 622 |  | - | E3/E4 |
| #4 | male | 63.3 | 8 | n.a. | PSP-RS | PSP, AGD, ARTAG, arteriosclerosis, mild Aβ pathology (Thal 2) | n.a. | 18.75 | 518 |  | Aβ: few diffuse plaques | n.a. |
| #5 | male | 61.1 | 5 | n.a. | PSP-RS | PSP, old frontal contusion, macro/microangiopathy | 1150 | 72.00 | 243 |  | - | E3/E3 |
| #6 | female | 73.9 | 8 | n.a. | PSP-RS | PSP, AGD | 1140 | 55.00 | 375 |  | - | E3/E3 |
| #7 | female | 87.4 | 13 | n.a. | PSP-CBS | PSP, LBD (brain stem type), CAA | 1060 | 79.17 | 335 |  | - | E2/E3 |
| #8 | female | 79.5 | 5 | cardiac arrest | PSP-RS | PSP, AGD | 1000 | 15.83 | 113 |  | - | E3/E3 |
| #9 | male | 68.8 | 5 | cardiac arrest | FTLD,  probably PSP | PSP, ischemic infarction of the left CA1 region, arteriosclerosis | 1340 | 89.08 | 116 |  | - | E3/E3 |
| #10 | male | 77.2 | 8 | bronchopneumonia, sepsis | PSP-RS | PSP, CAA, AD (B&B 3, Thal 1), ARTAG, arteriosclerosis, microinfarct of the left cerebellum | 1180 | 42.83 | 119 |  | Aβ: few plaques | n.a. |
| #11 | male | 75.7 | 8 | n.a. | FTD | PSP, AGD | n.a. | 97.52 | 246 |  | - | n.a. |
| #12 | male | 76.4 | 14 | aspiration pneumonia, subileus | PSP-RS | PSP | 1045 | 40.25 | 255 |  | - | n.a. |
| #13 | male | 75.8 | 5 | multiple organ failure | PSP-RS | PSP | 1440 | 40.82 | 477 |  | - | n.a. |
| #14 | male | 74.1 | 12 | n.a. | PSP-RS | PSP | 1200 | 11.58 | 675 |  | - | n.a. |
| #15 | male | 67.8 | 6 | cachexia | PSP-RS | PSP | 1460 | 37.50 | 195 |  | - | n.a. |
| #16 | male | 66.2 | 5 | cardiac and respiratory failure | PSP-RS | PSP | NA | 55.00 | 216 |  | - | n.a. |

**Supplemental Table 2–Overview of samples from the autoradiography cohort of patients with PSP.** y = years, LBD = Lewy body disease, ALS = amyotrophic lateral sclerosis, FTD = frontotemporal dementia, PSP-RS = progressive supranuclear palsy Richardson syndrome, nf = nonfluent, PPA = primary progressive aphasia, bv = behavioral variant, AD = Alzheimer’s disease, CAA = cerebral amyloid angiopathy, AGD = agyrophilic grain disease, ARTAG = aging-related tau astrogliopathy, B&B = Braak and Braak, TDP-43 = TAR DNA-binding protein 43, MND = motor neuron disease, PART = primary age-related tauopathy, Aβ = β-amyloid, APOE = apolipoprotein E, α-syn = alpha synuclein, FUS = fused in sarcoma, n.a. = not available, "-" = negative

**Supplemental Table 3**

| **Case** | **Demographics** | | | | **Diagnosis** | | **Autopsy determinants** | | |
| --- | --- | --- | --- | --- | --- | --- | --- | --- | --- |
|  | Sex | Age at  death (y) | Disease  duration (y) | Cause of death (death certificate) | Clinical  diagnosis | Autopsy  diagnosis [1] | Brain  weight (g) | Postmortem  delay (h) | Fixation  time (d) |
| #1 | male | 74.8 | 21 | n.a. | PDD/DLB | LBD (Braak stage 6, neocortical),  AD/PART (B&B stage 2, Thal 0)),  microangiopathy (Thal stage 3) | 1250 | 42.25 | 100 |
| #2 | male | 77.8 | 26 | n.a. | PD | LBD (Braak stage 6, neocortical), AD/PART (B&B stage 1, Thal 0) | 1575 | 124.47 | 75 |
| #3 | female | 74.5 | 42 | n.a. | PD | LBD (Braak stage 6, neocortical), AD (B&B stage 1, Thal 1) | 1280 | 22.08 | 307 |
| #4 | female | 79.7 | 12 | n.a. | PDD | LBD (Braak stage 6, neocortical), AD/PART (B&B stage 1, Thal 0) | n.a. | <37 | n.a. |

**Supplemental Table 3–Overview of samples from the autopsy cohort of patients with PD.** y = years, PD = Parkinson’s disease, PDD = Parkinson’s disease with dementia, AD = Alzheimer’s disease, LBD = Lewy body disease, B&B = Braak and Braak. n.a. = not available

1. Attems, J., et al., *Neuropathological consensus criteria for the evaluation of Lewy pathology in post-mortem brains: a multi-centre study.* Acta Neuropathologica, 2021. **141**(2): p. 159-172.
